# PhoPQ Regulates Quinolone and Cephalosporin Resistance Formation in Salmonella Enteritidis at the Transcriptional Level

**DOI:** 10.1128/mbio.03395-22

**Published:** 2023-05-15

**Authors:** Mengjun Hu, Yuyan Zhang, Xiaozhen Huang, Mu He, Jinyu Zhu, Zengfeng Zhang, Yan Cui, Shoukui He, Xianming Shi

**Affiliations:** a Department of Food Science & Technology, School of Agriculture & Biology, and State Key Laboratory of Microbial Metabolism, Shanghai Jiao Tong University, Shanghai, China; MedImmune

**Keywords:** two-component system, PhoPQ, antibiotic resistance, transcriptional regulation, antibiotic recognition

## Abstract

The two-component system (TCS) PhoPQ has been demonstrated to be crucial for the formation of resistance to quinolones and cephalosporins in *Salmonella* Enteritidis (*S.* Enteritidis). However, the mechanism underlying PhoPQ-mediated antibiotic resistance formation remains poorly understood. Here, it was shown that PhoP transcriptionally regulated an assortment of genes associated with envelope homeostasis, the osmotic stress response, and the redox balance to confer resistance to quinolones and cephalosporins in *S.* Enteritidis. Specifically, cells lacking the PhoP regulator, under nalidixic acid and ceftazidime stress, bore a severely compromised membrane on the aspects of integrity, fluidity, and permeability, with deficiency to withstand osmolarity stress, an increased accumulation of intracellular reactive oxygen species, and dysregulated redox homeostasis, which are unfavorable for bacterial survival. The phosphorylated PhoP elicited transcriptional alterations of resistance-associated genes, including the outer membrane porin *ompF* and the aconitate hydratase *acnA*, by directly binding to their promoters, leading to a limited influx of antibiotics and a well-maintained intracellular metabolism. Importantly, it was demonstrated that the cavity of the PhoQ sensor domain bound to and sensed quinolones/cephalosporins via the crucial surrounding residues, as their mutations abrogated the binding and PhoQ autophosphorylation. This recognition mode promoted signal transduction that activated PhoP, thereby modulating the transcription of downstream genes to accommodate cells to antibiotic stress. These findings have revealed how bacteria employ a specific TCS to sense antibiotics and combat them, suggesting PhoPQ as a potential drug target with which to surmount *S.* Enteritidis.

## INTRODUCTION

*Salmonella* Enteritidis (*S.* Enteritidis) is responsible for 65% of nontyphoidal Salmonella infections with symptoms of nausea, emesis, and diarrhea (1, 2). Quinolones and cephalosporins are the drugs of choice for treating pediatric patients who have complicated Salmonella infections (3). However, the prevalence of *S.* Enteritidis isolates with resistance to multiple antimicrobials, including quinolones and cephalosporins, makes this serotype a serious public health concern (4–7). Resistance to quinolones is commonly mediated by mutations that reduce the affinity of quinolones to topoisomerase (8). Cephalosporin resistance is usually achieved by β-lactamases that degrade and inactivate cephalosporins (9). In addition, the genes encoding the multidrug efflux pump and the outer membrane (OM) porin also contribute to resistance by controlling the intracellular levels of drugs (9–11). Despite a set of resistance determinants being identified, it is still unclear how these determinants are regulated by Salmonella to deal with the stress posed by quinolones and cephalosporins.

Two-component systems (TCSs) are among the key elements through which bacteria sense and respond to environmental changes (12). A canonical TCS consists of an inner membrane imbedded histidine sensor kinase (HK) and a cytosolic response regulator (RR). When HK is activated by environmental stresses, it auto-phosphorylates and then transfers phosphoryl groups to its cognate RR for activation (13). The activated RR binds specifically to the promoters of target genes to regulate their transcription, thereby initiating cellular responses (14). A series of TCSs from various species have been reported to be associated with antibiotic resistance, including aminoglycoside and β-lactam resistance-associated CpxAR (15), drug efflux-associated EvgSA (16), β-lactam resistance-associated VbrKR and CreBC (17, 18), as well as polymyxin B resistance-associated PmrAB and PhoPQ (19). Collectively, TCSs represent a mechanism that is favorable for bacteria to survive under adverse environments.

PhoPQ is a conserved TCS that is involved in low-magnesium adaptation, virulence expression, and polymyxin resistance in Gram-negative pathogens (20). PhoQ, the dimeric HK of this system, contains a periplasmic sensor domain, a transmembrane (TM) domain, and three cytosolic domains. The dimeric sensor domain and (or) the TM domain adopt different conformations after sensing stimuli, such as low divalent cations (21), an acidic pH (22), an osmotic upshift (23), or the presence of cationic antimicrobial peptides (CAMPs) (24), to yield an autophosphorylated PhoQ. The phosphorylated PhoQ leads to a phosphorylated RR PhoP that binds to the promoters of target genes at the heptanucleotide sequence (G/T)GTTTA(A/T), thereby enabling coordinated changes in global gene expression profiles (25, 26). The regulons triggered by PhoPQ vary in a species-dependent manner among Salmonella Typhimurium (*S.* Typhimurium), E. coli, Pseudomonas aeruginosa (P. aeruginosa), Shigella flexneri, Yersinia pestis, and Mycobacterium tuberculosis (27–34). In our previous work, functional defects in PhoPQ in various *S.* Enteritidis isolates led to a frequent 2 to 8-fold decrease in minimal inhibitory concentration (MIC) values against quinolones and cephalosporins (35). However, the regulatory mechanism underlying the PhoPQ-mediated antibiotic resistance in *S.* Enteritidis remains poorly understood.

Here, we reported a previously unidentified regulatory role of the PhoPQ system that allows for the recognition of antibiotics and governs the quinolone and cephalosporin resistance pathways in *S.* Enteritidis. Via a strand-specific RNA-sequencing (RNA-seq) analysis, we assessed the impact of PhoPQ on the global transcription profile of *S.* Enteritidis treated by nalidixic acid and ceftazidime, which are a representative quinolone and cephalosporin, respectively. Notably, we identified the critical genes and progress pathways regulated by PhoPQ and demonstrated that nalidixic acid and ceftazidime molecules could be sensed by directly binding to PhoQ. This work will contribute to a better understanding of antibiotic resistance mechanisms as well as provide a basis upon which to combat antibiotic resistance in *S.* Enteritidis.

## RESULTS

### A comparative transcriptomic analysis reveals the differentially expressed genes regulated by PhoP in *S.* Enteritidis.

**FIG 1:**
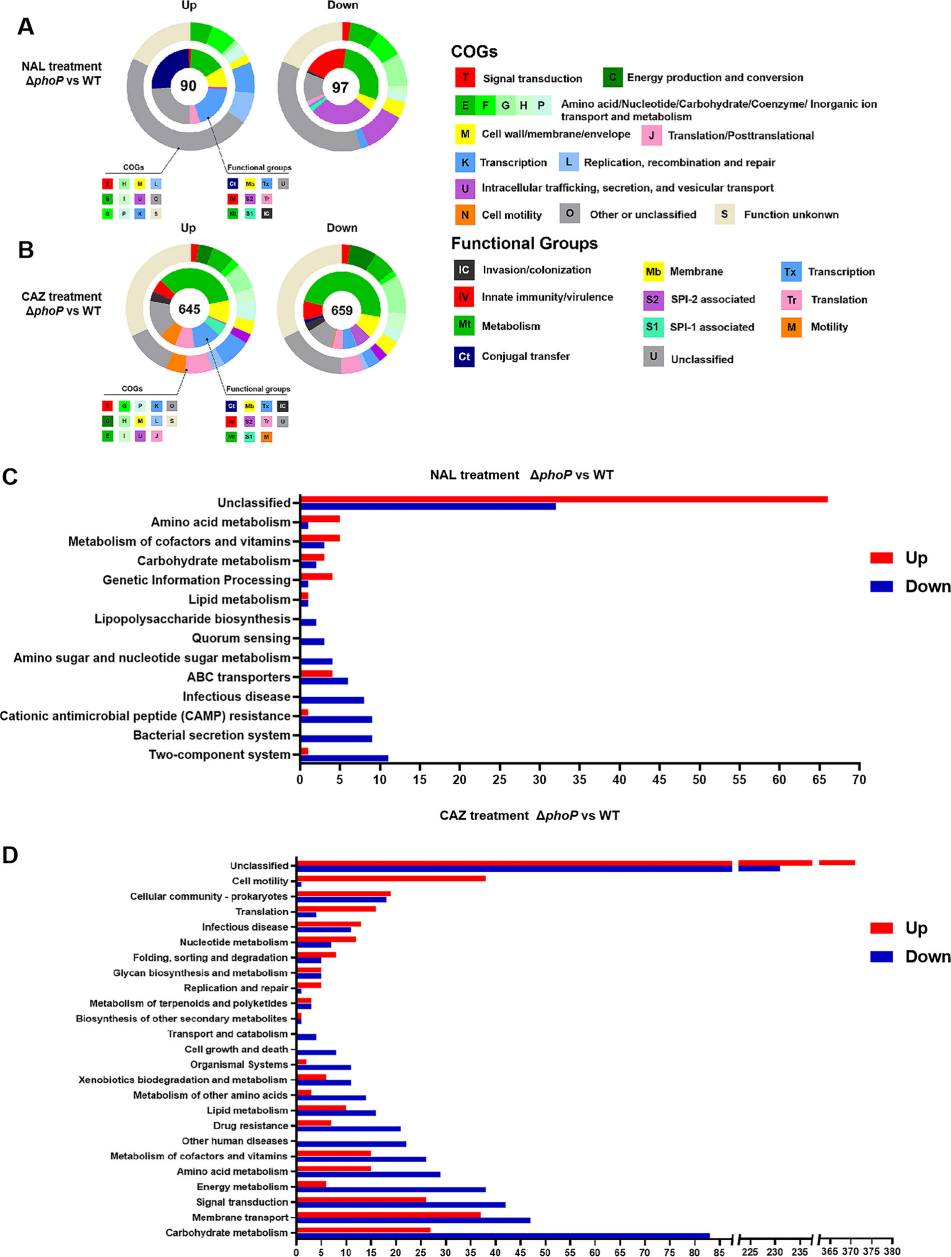
A transcriptomic analysis revealed global gene regulation by PhoP in *S.* Enteritidis. (A and B) The DEGs identified (Δ*phoP* versus WT) under the pressure of NAL (A) or CAZ (B) were clustered in terms of functional groups (inner ring) and COG categories (outer ring). The numbers of the DEGs are indicated in the circle centers. (C and D) KEGG annotation revealed the numbers of the DEGs under the pressure of NAL (C) or CAZ (D), enriched into various pathways.

To test the regulatory roles of *S.* Enteritidis PhoPQ in its global gene transcription to combat nalidixic acid (NAL) and ceftazidime (CAZ), the differentially expressed genes (DEGs) in a *phoP* deletion mutant, compared with the wild-type (WT), were identified via RNA-seq analysis. It was found that a total of 90 upregulated and 97 downregulated DEGs were identified under NAL pressure, whereas 645 upregulated and 659 downregulated DEGs were identified under CAZ pressure (Fig. 1). The RNA-seq data were verified via reverse transcription-quantitative PCR (RT-qPCR) assays (Fig. S1), which showed that the consistency levels between the RT-qPCR and RNA-seq results were 100% and 93.75% under NAL and CAZ conditions, respectively (Fig. S1).

10.1128/mbio.03395-22.2 FIG S1 RT-qPCR validation of RNA-seq data. (A) Agarose gel for RNA samples. Lanes 1 to 3, WT cells under nalidixic acid conditions at 8 h. Lanes 4 to 6, Δ*phoP* cells under nalidixic acid conditions at 8 h. Lanes 7 to 9, WT cells under ceftazidime conditions at 8 h; Lanes 10 to 12, Δ*phoP* cells under ceftazidime conditions at 8 h. All RNA samples meet the requirements of integrity. (B and C) RT-qPCR. The consistency levels between the RT-qPCR and RNA-seq results were 100% and 93.75% under nalidixic acid (B) and ceftazidime conditions (C), respectively. NP, nalidixic acid treated Δ*phoP* cells; CP, ceftazidime treated Δ*phoP* cells; NWT, nalidixic acid treated WT cells; CWT, ceftazidime treated WT cells. *16S rDNA* was used as a reference gene. The mean log_2_-fold change (FC) in the transcription of genes in three independent RT-qPCR experiments was plotted against the respective log_2_FC that was determined via RNA-seq. Download FIG S1, TIF file, 5.3 MB. Copyright © 2023 Hu et al. 2023 Hu et al. https://creativecommons.org/licenses/by/4.0/ This content is distributed under the terms of the Creative Commons Attribution 4.0 International license.

To gain insights into the metabolic pathways that are influenced by PhoP, the identified DEGs were assigned to functional classes and COG categories. It was demonstrated that 187 DEGs under NAL pressure were related to the metabolism, membrane, transcription, genomic islands, innate immunity/virulence, and conjugal transfer categories (Fig. 1A). In comparison, the CAZ pressure led to an increased number of DEGs in association with the metabolism and membrane categories as well as those associated with translation and motility, which were not observed under NAL pressure (Fig. 1B). These DEGs were generalized into three strikingly regulated aspects: (i) innate immunity defense, (ii) substrate transport and metabolism progress, and (iii) cell envelope homeostasis.

A KEGG cluster analysis (Fig. 1C and D) revealed an array of significantly changed pathways under NAL pressure, including amino acid/carbohydrate/cofactor and vitamin metabolism, two-component systems, bacterial secretion systems, CAMP resistance, quorum sensing, and lipopolysaccharide (LPS) biosynthesis. In addition, CAZ pressure resulted in significant changes in the pathways related to carbohydrate metabolism, signal transduction, drug resistance, lipid metabolism, etc. Intriguingly, it was found that some previously unidentified pathways and genes were involved in DNA replication and repair, ABC transporters, peptidoglycan synthesis, and membrane transport. Here, we integrated the correlative pathways and genes under NAL/CAZ pressures, and we analyzed the largely affected DEGs in depth. These DEGs were responsible for envelope integrity, the osmotic stress response, and the redox balance.

### PhoP regulates the envelope integrity in *S.* Enteritidis.

**FIG 2:**
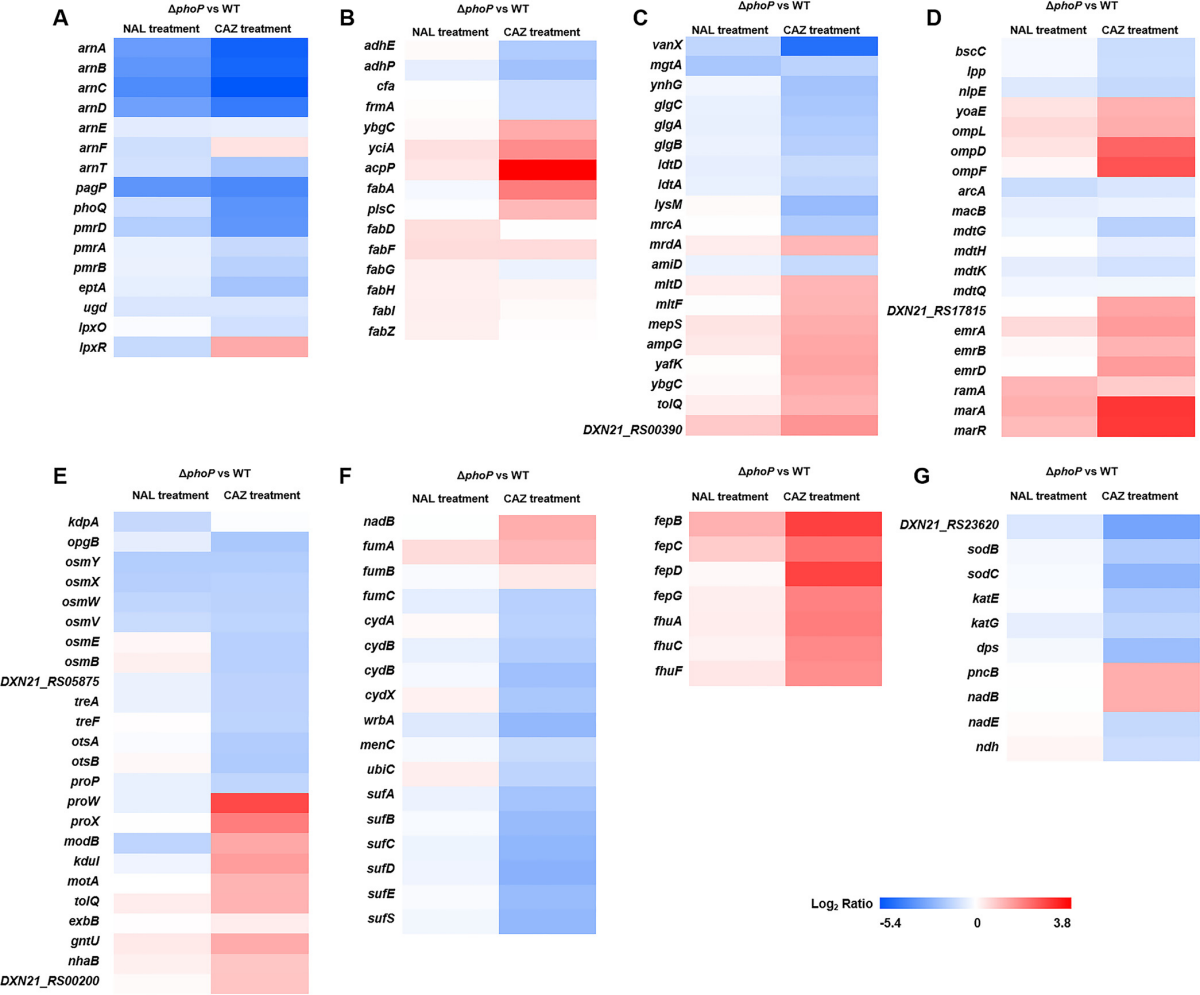
Heat map depicting the relative transcription levels of the genes from various pathways regulated by PhoP under antibiotic pressures. (A) LPS modification. (B) Fatty acid synthesis and degradation pathways. (C) Peptidoglycan synthesis, cross-linking, and degradation; (D) Membrane-related innate genes. (E) Osmotic stress response. (F and G) Redox balance, including ROS formation and scavenging, quinone synthase, Fe-S cluster assembly, the iron ion ABC transporter genes, and the NADH-related electron transport chain. Each row represents an individual gene. The relative transcription level (Δ*phoP* versus WT) is indicated as a Log_2_-fold change (FC) that is labeled in the corresponding grid. The scale of this heat map is given as the log_2_ FC, ranging from −5.4 (blue) to 3.8 (red).

**FIG 3:**
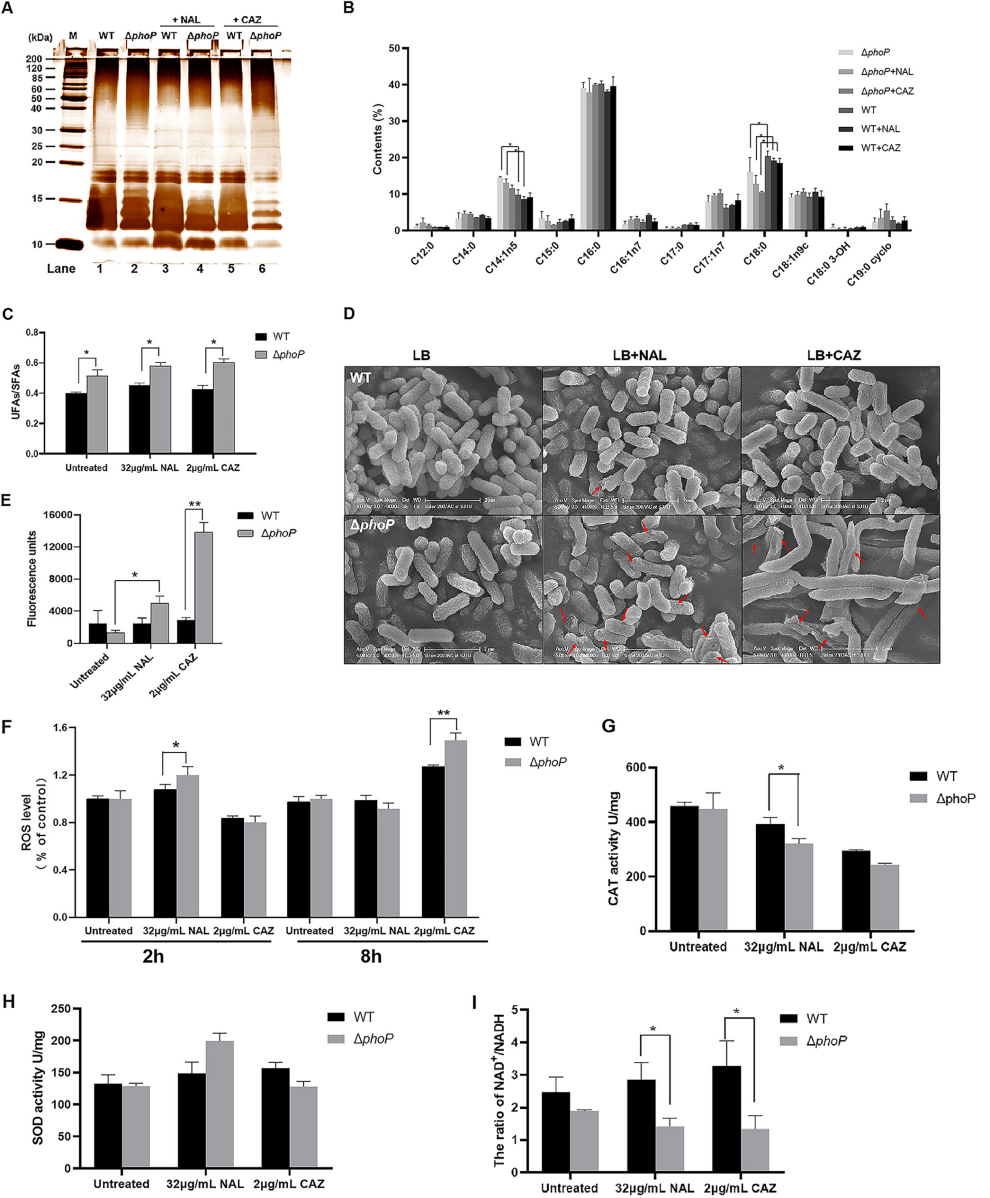
The deletion of *phoP* altered the physiological behaviors of *S.* Enteritidis. (A) Silver staining of membrane lipopolysaccharide extraction. The dark aggregated bands below 20 kDa suggest the presence of smaller oligosaccharide fragments and fatty acid chains released from the core-polysaccharide and lipid-A region of LPS. “M” is the protein marker. (B) GC-MS analyses revealed the cell membrane fatty acid composition with the abundance of each fatty acid (C) as well as the calculated SFAs/UFAs ratios. (D) TEM observation of cellular morphology and membrane damage. (E) NPN uptake assay. (F) Cellular ROS level. (G) CAT activity. (H) SOD activity. (I) Cellular NAD^+^/NADH ratios. All experiments were performed in at least three biological replicates. Error bars indicate the standard deviation of 3 to 5 technical replicates. Statistical significance: *, *P* < 0.01.

The expression profiles of DEGs related to bacterial envelope integrity, including LPS modification, fatty acid synthesis, and degradation pathways, peptidoglycan synthesis, cross-linking and degradation, and membrane-related innate proteins were analyzed in this study. The transcription of the genes involved in LPS modification in Δ*phoP* was virtually downregulated under both antibiotic pressures (Fig. 2A). This is exemplified by the UDP-glucose 6-dehydrogenase gene (*ugd*) and the lipid A hydroxylase gene (*lpxO*), the products of which modify lipid A phosphates with 4-amino-4-deoxy-l-arabinose or hydroxylation acyl chains. In addition, the transcription of the PmrAB-targeted *arn* operon (aminoarabinose), *eptA* (phosphoethanolamine), and *pagP* (palmitoyltransferase) that function to stabilize the membrane by producing more negatively charged LPS was also downregulated, implying interrupted LPS in Δ*phoP*. The results from a silver staining assay of membrane LPS extracts supported the RNA-seq data, meaning that the deletion of *phoP* resulted in a decrease in production of LPS with fewer and shallower stained ladders and that this deficiency was exacerbated under the pressure of NAL or CAZ (Fig. 3A). These data suggested positive regulatory impacts of PhoPQ on LPS production and modification.

Under CAZ pressure, the transcription of the genes involved in fatty acid synthesis (*yciA*, *fabA*, *ybgC*, and *acpP*, etc.) and phospholipid synthesis (*plsC*) was significantly upregulated (1.04 to 3.71-fold), whereas those related to cyclopropane fatty acyl phospholipid synthesis (*cfa*) and fatty acid degradation (*adhP*, *adhE*, and *frmA*) were significantly downregulated (1.08 to 1.99-fold) (Fig. 2B). The consistent variations in the expression of these genes, albeit to a less significant degree, were also observed under NAL pressure (Fig. 2B). These identified DEGs hinted at a fluctuant composition of membrane fatty acids in Δ*phoP*, and this was supported by the fatty acids analysis, in which Δ*phoP* exhibited significant a decrease in the saturated fatty acid (SFAs) C18:0 and an increase in the unsaturated fatty acids (UFAs) C14:1n5 and C17:1n7, with a significantly higher ratio of UFAs to SFAs (UFAs/SFAs) (51.49% to 60.41%) than that observed in the WT (39.92% to 45.12%) (Fig. 3B and C). The UFAs/SFAs ratio was a predominant parameter with which to evaluate membrane fluidity in bacteria, and bacterial cells with lower membrane fluidity usually exhibited higher resistance to environmental stresses (36). Hence, the altered fatty acids with a higher UFAs/SFAs ratio in Δ*phoP* might, to some degree, explain the increased susceptibility to quinolones and cephalosporins.

The deletion of *phoP* led to the downregulation of the genes involved in peptidoglycan (PG) synthesis and cross-linking (Fig. 2C), including the glycogen synthase operon *glg*, which is responsible for the elongation of glycogen, the D, d-transpeptidase gene *vanX* for the primary cross-linking of PG with d-Ala^4^-meso-DAP^3^, the L, d-transpeptidase genes *ldtA*, *ldtD*, and *ynhG* for the meso-DAP^3^-meso-DAP^3^ cross-links (37–39), and the glycosyltransferase gene *mgtA*, which is involved in PG polymerization. Additionally, the PG hydrolysis-related *mltD*, *mltF*, *mepS*, *ampG*, *yafK*, and *DXN21-RS00390* genes were upregulated (1.08 to 1.55-fold) under CAZ pressure, with the corresponding variations, albeit to a lesser degree, being observed under NAL pressure (Fig. 2C). These results indicated that the deletion of *phoP* interferes with PG synthesis and exacerbates PG hydrolysis, which might lead to a poorly cross-linked PG layer with weakened mechanical strength. The scanning electron microscopic (SEM) results showed that a considerable number of lysed cells were found in Δ*phoP* in the presence of NAL or CAZ (Fig. 3D). Of note, the WT cells retained their characteristic rod shape, with an average length of 1.6 μm (ranging from 1.35 to 2.25 μm for 20 cells examined), under both treatments, whereas the Δ*phoP* cells were largely elongated (with the length being hard to examine) under the CAZ treatment, suggesting uncoordinated elongation and division. Given the role of coordinated division with PG synthesis-mediated elongation in cell shape determination (40), the downregulated cell division-associated genes *zapA*, *murG*, *ftsL*, *ftsW*, and *lpoB*, as well as the upregulated Tol-Pal system (which is required for proper PG remodeling at the division site) genes *ybgC* and *tolQ*, might be responsible for the abnormal cell morphologies (Fig. S2).

10.1128/mbio.03395-22.3 FIG S2 Heat map of genes involved in cell division and elongation, SPI-2, and energy mechanism pathways. (A) Cell division and elongation. (B) SPI-2. (C) Energy mechanism. The scale of this heat map was given as the log_2_ FC value, ranging from −5.4 (blue) to 3.8 (red). Download FIG S2, TIF file, 6.4 MB. Copyright © 2023 Hu et al. 2023 Hu et al. https://creativecommons.org/licenses/by/4.0/ This content is distributed under the terms of the Creative Commons Attribution 4.0 International license.

In Δ*phoP*, cell membrane-related genes, including the glucan transportation gene *bcsC*, the envelope stress response gene *nlpE*, the OM-peptidoglycan cross-linking gene *lpp*, and the efflux pump genes *arcA*, *macB*, and *mdt* operon were downregulated, whereas the OM porin genes *ompF*, *ompD*, and *ompL* were upregulated (Fig. 2D), implying more admission to drugs through OM porins, accompanied with inefficiencies in expelling them via efflux pumps. The NPN (1-n-phenylnapthylamine) uptake assays also supported the role of PhoPQ in the limitation of envelope permeability under antibiotic pressure, as the deletion of *phoP* rendered the cells exposed to CAZ/NAL with a stronger NPN fluorescence intensity (Fig. 3E). Altogether, the PhoP regulator functions in the maintenance and modification of the bacterial envelope by transcriptionally regulating multiple associated genes.

### PhoP regulates the osmotic stress response genes in *S.* Enteritidis.

The RNA-seq data revealed that the ABC transporter pathway is regulated by PhoP. Under NAL/CAZ pressure, the deletion of *phoP* led to the transcriptional downregulation of the genes that encode K^+^ transport (*kdpA*), osmoprotectants (*proP*), and ABC-type osmotically inducible proteins (*osmV*, *osmX*, *osmY*, and *osmW*) as well as the upregulation of the genes that encode Na^+^-Ca^2+^/H^+^ antiporters (*nhaB*, *gntU*, and *DXN21_RS00200*) (Fig. 2E), of which the upregulated antiporters would cause a low intracellular accumulation of metal ions. In addition, under CAZ pressure, the genes that encoded osmotically inducible proteins (*osmB* and *osmE*) and were involved in trehalose synthesis and utilization (*otsA*, *otsB*, *treA*, *treF*, and *DXN21_RS05875*) were also downregulated (Fig. 2E). Gram-negative bacteria usually accumulate K^+^, nonpolar and facultative ionic solutes, trehalose, and glycine betaine to tackle osmotic stress (41). Therefore, PhoP activation, mediated by exposure to antibiotics, may improve the transport of substrates via the regulation of the genes that are related to osmotic stress responses.

### PhoP regulates the redox balance in *S.* Enteritidis.

The genes that are responsible for the redox balance were shown to be differentially expressed in Δ*phoP* and WT under both NAL and CAZ pressure, including reactive oxygen species (ROSs) formation and scavenging, quinone synthase, Fe-S cluster assembly, the iron ion ABC transporter, and the NADH-related electron transport chain (Fig. 2F and G). In the Δ*phoP* cells, the genes of *nadB* and *fumA* were upregulated, whereas the genes related to quinone oxidoreductase (*wrbA*), menaquinone synthase (*menC*), ubiquinone synthase (*ubiC*), and cytochrome bd oxidase (*cydABX*) were downregulated (Fig. 2F), implying the accumulation of H_2_O_2_ in cells lacking PhoP. The genes responsible for the Fe-S cluster assembly (*sufABCDES*) were downregulated, whereas the iron ion ABC transporter genes (*fepBCDG* and *fhuACF*) were upregulated, implying an increase in free iron that was readily oxidized by H_2_O_2_ to trigger the Fenton reaction (Fig. 2F). This was supported by the significantly higher ROS levels that were observed in the Δ*phoP* cells that were exposed to both antibiotics (Fig. 3F).

The decreased transcription of the catalase (CAT) genes *katE* and *katG*, the superoxide dismutase (SOD) genes *DXN21_RS23620*, *sodB*, and *sodC*, which function to scavenge intracellular H_2_O_2_ or superoxide, as well as the decreased transcription of *dps*, whose product acts to mineralize and store iron ions in a bioavailable and nontoxic form and protect DNA during oxidative stress, were observed in the Δ*phoP* cells that were exposed to both antibiotics (Fig. 2G). These DEGs indicated that PhoP has a role in antioxidation pathways, and this result was supported by lower CAT activities and SOD enzymes being observed in the Δ*phoP* cells that were exposed to antibiotics (Fig. 3G and H). Although the Δ*phoP* cells that were exposed to NAL had higher SOD activities, this was presumably due to the complementary effects of the total SOD enzymes, other than SodB and SodC.

An equivalent cofactor NADH that functions as a distinct electron carrier can be oxidized to NAD^+^ to promote the redox balance. In this study, the upregulated NAD^+^ biosynthesis genes *nadB* and *pncB* as well as the downregulated ammonia-dependent NAD^+^ synthetase gene *nadE* under CAZ pressure suggested a role of PhoP in NAD^+^ production, even though the transcriptional variations in these genes were not significant when exposed to NAL (Fig. 2G). The role of PhoP was supported by the significantly decreased NAD^+^/NADH ratio in the Δ*phoP* cells that were exposed to antibiotics (Fig. 3I), indicating that the deletion of *phoP* disturbed the cellular redox balance. Additionally, the NADH dehydrogenase II-encoding *ndh* that functions to convert NADH to NAD^+^ was also downregulated, which might lead to the accumulation of NADH and was partially responsible for the decreased NAD^+^/NADH ratio. Taken together, these data suggested that PhoP regulates cellular redox homeostasis and limits the accumulation of ROS in response to antibiotic stress, which helps *S.* Enteritidis combat the antibiotics.

### PhoP directly regulates the *ompF* and *acnA* genes in *S.* Enteritidis.

**FIG 4:**
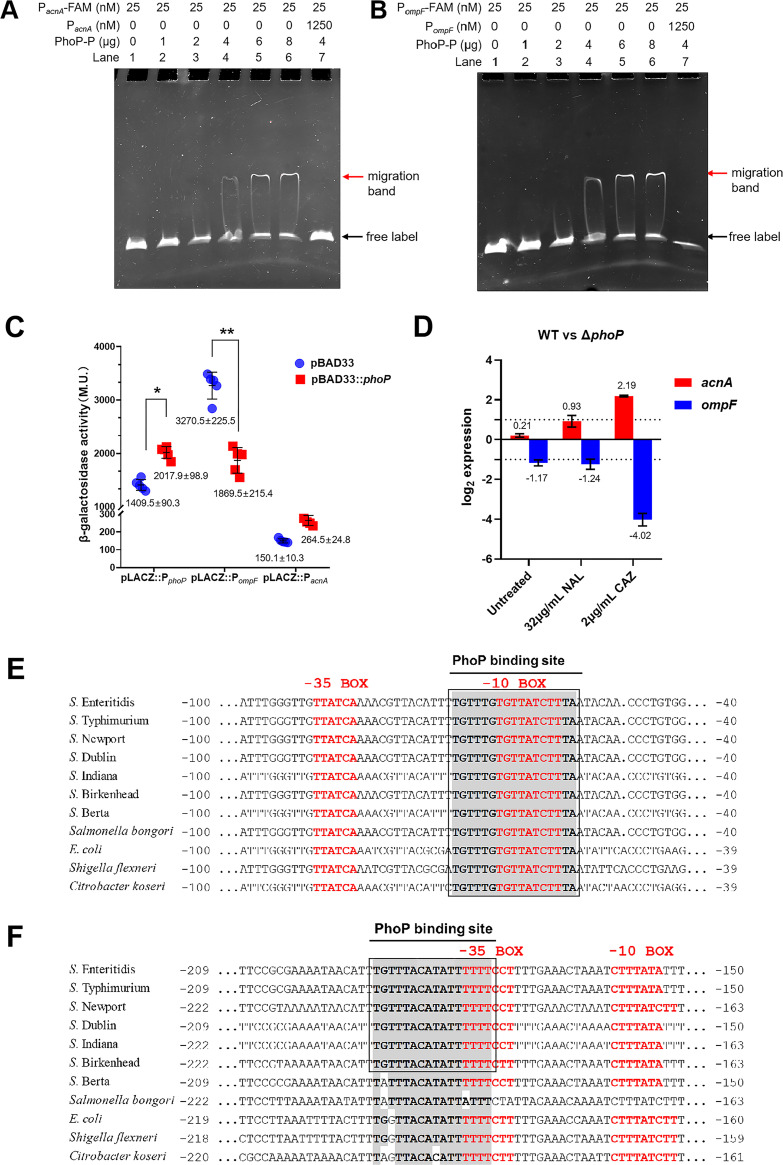
The promoters of *acnA* and *ompF* were directly targeted by PhoP. (A and B) An EMSA revealed the binding capacity of PhoP-P to the *acnA* (A) and *ompF* (B) promoter regions. Lanes 1 to 6, FAM-labeled target gene promoter (P*_acnA_*-FAM/P*_ompF_*-FAM), incubated with different amounts of PhoP-P (0, 1, 2, 4, 6 and 8 μg); lane 7, 50× unlabeled target gene promoter probe (P*_acnA_*/P*_ompF_*), competed with labeled probes. For each lane, 0.5 μg of poly(dI-dC) was added to decrease nonspecific binding. (C) β-galactosidase activity. The LacZ (β-galactosidase) activity of strains harboring P*_acnA_*/P*_ompF_*/P*_phoP_* promoter-*lacZ* fusions with an arabinose-inducible expression of PhoP was measured after incubation for 5 h in the presence of arabinose (0.2%). The data were presented as a scatterplot, showing the mean and standard error of three independent experiments. (D) Relative expression levels of *acnA* and *ompF* in WT cells, compared with those in Δ*phoP* cells under different conditions. (E and F) Alignment of the *acnA* (E) and *ompF* (F) promoter regions from different species. The (G/T)GTTTA(A/T) direct repeats for PhoP binding are boxed, and the proposed −35 and −10 elements are marked in red.

To identify the genes directly regulated by PhoP in *S.* Enteritidis, the putative PhoP-binding motifs (5′-[T/G] GTTTA-N5-[T/G] GTTTA-3′) in *S.* Typhimurium and E. coli were mapped to the promoter regions of 1,481 DEGs (1,304 and 187 for CAZ and NAL, respectively). As a result, 68 PhoP-targeted candidates were screened (Fig. S3). Eight of them (*vanX*, *mgtA*, *mgtC*, *slyB*, *pagP*, *sseL*, *pgtE*, and *yoaE*) were reported to be PhoP-targeted genes in previous studies (25, 33, 42, 43). To figure out the PhoPQ-targeted genes that were responsible for antibiotic resistance, genes involved in envelope homeostasis (*osmY*, *cfa*, *ompF*, *yoaE*, and *lpp*) and carbon/nucleotide metabolism (*csrA*, *acnA*, and *rbsK*) were selected for the following tests. The His-tagged PhoP protein was expressed in E. coli (Fig. S4), and its ability to bind to the promoters of the candidate genes was tested via electrophoretic mobility shift assays (EMSAs). As a result, the addition of phosphorylated PhoP (PhoP-P) blocked the mobility shift of the fragments upstream of *ompF* and *acnA* in a concentration-dependent manner (Fig. 4A and B). These fragments were a 221-bp FAM-labeled *ompF* promoter probe (P*_ompF_*-FAM) and a 231-bp *acnA* promoter probe (P*_acnA_*-FAM), suggesting a capacity of PhoP-P to bind to the promoter of *ompF* and *acnA*. Additionally, this binding was specific, as it was completely abolished by a 50× unlabeled *ompF* or *acnA* promoter probe (P*_ompF_* and P*_acnA_*) (Fig. 4A and B, lane 7).

10.1128/mbio.03395-22.4 FIG S3 Prediction of the promoters regulated by the PhoP. (A) The sequence logo of the PhoP box motif. (B) Functional classification of the 68 putative PhoP target genes. The putative PhoP binding sites in the promoter region were restricted to approximately 600 bp before the start codon with at most 2 unmatched nt. a, metabolism; b, envelope homeostasis; c, virulence, chemotaxis, and motility; d, unknown function. Download FIG S3, TIF file, 1.5 MB. Copyright © 2023 Hu et al. 2023 Hu et al. https://creativecommons.org/licenses/by/4.0/ This content is distributed under the terms of the Creative Commons Attribution 4.0 International license.

10.1128/mbio.03395-22.5 FIG S4 Expression and purification of His-tagged PhoP in E. coli BL21. (A) Cloning of the *phoP* gene fragment. M, 100 bp marker. (B) SDS-PAGE and Western blot analysis of the bacterial lysate. Lane 1, the BL21 strain with empty plasmid. Lanes 2 to 5, the BL21 strain with the pET28a-*hisphoP* plasmid. Primary antibody, mouse anti-His-tag monoclonal antibody. Secondary antibody, goat anti-mouse IgG polyclonal antibody. M, protein marker. (C) Purification of His-tagged PhoP protein via Ni-NTA column. Lane 1: bacterial whole protein after sonication. Lanes 2 to 18, different gradients of the imidazole (20mM, 50mM, 100mM, and 250mM) flux protein. M, protein marker. Download FIG S4, TIF file, 3.1 MB. Copyright © 2023 Hu et al. 2023 Hu et al. https://creativecommons.org/licenses/by/4.0/ This content is distributed under the terms of the Creative Commons Attribution 4.0 International license.

To assess the regulatory effects of PhoP on *ompF* and *acnA*, we constructed transcriptional fusions by combining the native promoter of each candidate with a *lacZ* (encoding β-galactosidase) reporter. The resulting pLACZ-P*_phoP_*, pLACZ-P*_ompF_*, or pLACZ-P*_acnA_* was transformed into E. coli DH5α with an arabinose inducible expression of the *phoP* vector (pBAD33-*phoP*) or an empty vector (pBAD33). The β-galactosidase activity of the DH5α introduced with pLACZ, pBAD33, [pLACZ]:[pBAD33], pBAD33-*phoP*, or [pLACZ]:[pBAD33-*phoP*] was monitored to avoid background noises. As a positive control, the β-galactosidase activity was significantly increased in DH5α with [pBAD33-*phoP*]:[pLACZ-P*_phoP_*], in comparison to DH5α with [pBAD33]:[pLACZ-P*_phoP_*] (Fig. 4C). The expression of *phoP* induced by arabinose significantly decreased the β-galactosidase activity of the cells with a [pLACZ-P*_ompF_*] reporter (*P* < 0.01) and insignificantly increased that of the cells with [pBAD33-*phoP*]:[pLACZ-P*_acnA_*] (Fig. 4C), indicating that: (i) PhoP can regulate the transcription of *ompF* and *acnA* via their own promoters and (ii) PhoP plays reverse roles in the regulation of *ompF* and *acnA*. To explain the insignificant regulatory function of PhoP on *acnA*, we compared the transcription level of the *acnA* gene in the WT with those of the Δ*phoP* cells under different conditions. RT-qPCR results suggested that the presence of PhoP did not lead to an increased expression of *acnA* in cells under no antibiotic pressure but did under the pressure of NAL or CAZ (Fig. 4D). Therefore, other cofactors (e.g., sigma factors, c-di-GMP, ppGpp) might be required for PhoP to regulate the transcription of *acnA*.

A comparative analysis on the *acnA* promoter region in several species revealed that this identified PhoP binding site “TGTTTGTGTTATCTTTA” was highly conserved across Salmonella, including Salmonella bongori (S. bongori), *S.* Enteritidis, *S.* Typhimurium, *S.* Newport, *S.* Dublin, *S.* Indiana, *S.* Birkenhead, and *S.* Berta, as well as E. coli, Shigella flexneri, and Citrobacter koseri, indicating that this regulatory mechanism was conserved across these species (Fig. 4E). Surprisingly, the *ompF* promoter region was not conserved in S. bongori or in E. coli, Citrobacter koseri, and Shigella flexneri, implying that the identified PhoPQ regulatory mechanism on the *ompF* gene might be specific to S. enterica (Fig. 4F). These results provided clues regarding how bacteria evolve regulons to control gene transcription in the presence of antibiotics.

### Quinolones and cephalosporins lead to phosphorylated PhoQ.

**FIG 5:**
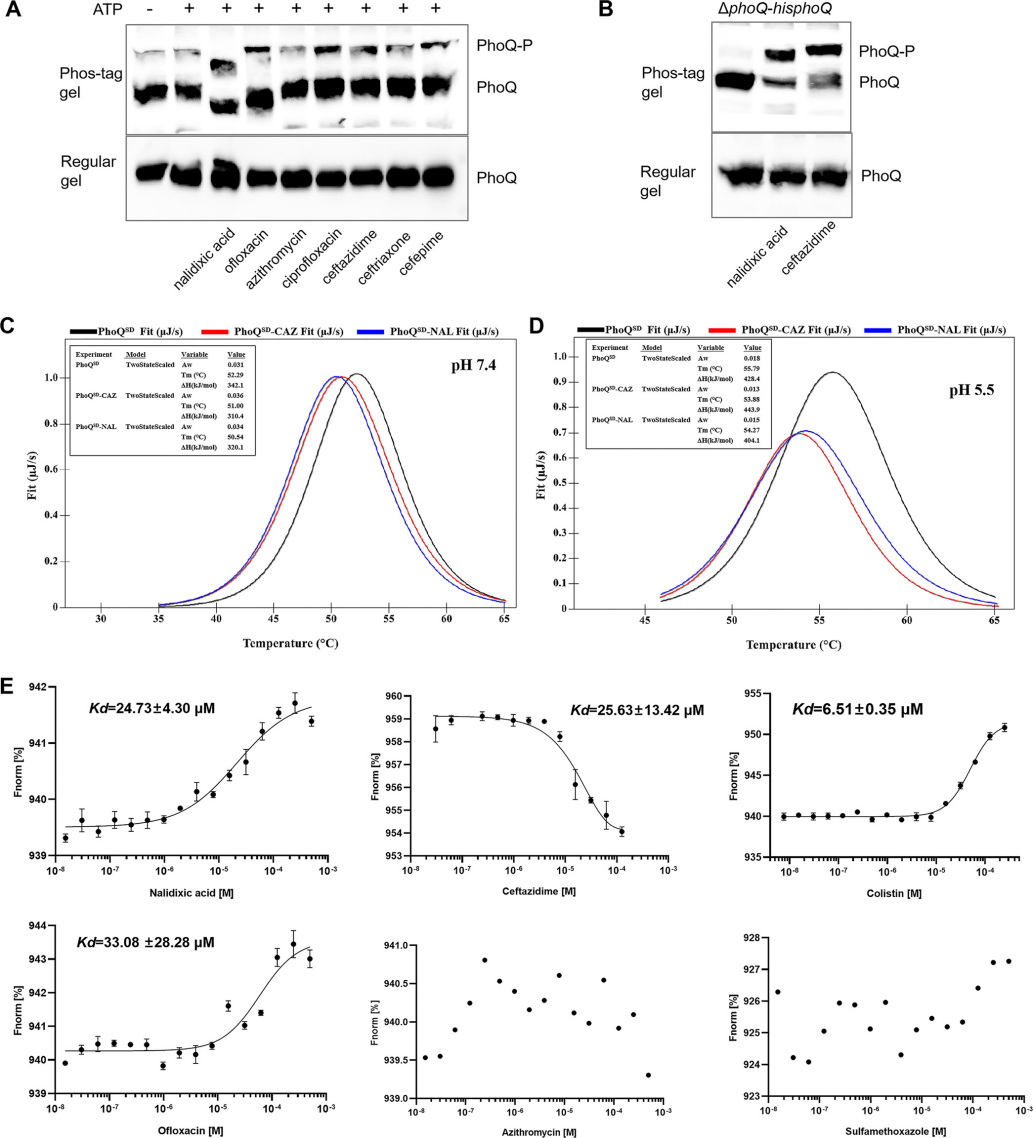
Analysis of PhoQ phosphorylation and its interaction with antibiotics. (A) *In vitro* phosphorylation assay. Membrane extracts from E. coli DH5α expressing 6×His-tagged PhoQ were treated with NAL, ciprofloxacin, ofloxacin, azithromycin, CAZ, ceftriaxone, and cefepime (lanes 3 to 9) or were left without treatment (lanes 1 to 2). No addition of ATP was used as a negative control (lane 1). Following treatment, each sample was resolved in the Phos-tag gel (upper panel) or regular gel (lower panel) and blotted with anti-His antibody. (B) *In vivo* phosphorylation assay. The whole-cell lysates of *S.* Enteritidis Δ*phoQ*-*hisphoQ* strains without treatment (lane 1) or those treated with NAL (lane 2) or CAZ (lane 3) were obtained for analysis; (C and D) DSC thermograms of PhoQ^SD^ and its mixture with NAL/CAZ in neutral pH (C) or under slightly acidic conditions (D). (E) MST determination of the affinity of PhoQ^SD^ with antibiotics, including NAL, CAZ, colistin, ofloxacin, azithromycin, and sulfamethoxazole. Antibiotics were titrated against fixed concentrations of labeled RED-tris-NTA-PhoQ^SD^ (100 nM). The *K_d_* fitting curve derived from the raw data was measured at 25°C, using 80% LED power and medium MST power.

To further investigate whether PhoQ is sufficient to respond to quinolones and cephalosporins, we isolated membrane extracts from an E. coli DH5ɑ strain expressing His-tagged PhoQ. The membrane extracts were treated with both antibiotics and ATP *in vitro*, and PhoQ phosphorylation was assessed via a Phos-tag assay. A shifted band of phosphorylated PhoQ (PhoQ-P) was observed when the membrane extract was treated with quinolones (nalidixic acid, ciprofloxacin, and ofloxacin), cephalosporins (ceftazidime, ceftriaxone, and cefepime) or ATP; the intensity of such a shifted band was much lower when treated with azithromycin or when without antibiotics (Fig. 5A, lanes 1 and 5). *In vivo*, we grew an *S.* Enteritidis Δ*phoQ* mutant (Fig. S5), and then the Δ*phoQ*-*hisphoQ* compensatory strain was constructed for a Phos-tag assay. In the presence of NAL or CAZ, a shifted band of PhoQ-P was detected in the whole-cell lysate of Δ*phoQ*-*hisphoQ* (Fig. 5B), suggesting that PhoQ was phosphorylated. Taken together, these results indicated that the presence of quinolones or cephalosporins led to phosphorylated PhoQ.

10.1128/mbio.03395-22.6 FIG S5 Schematics of *phoQ* knockout. (A) Schematics of *phoQ* deletion. The black lines and parts in the gene indicate the upstream flank (UF) and the downstream flank (DF). For in-frame mutagenesis, the mutation maintained a fraction of the codons of the gene. Thin arrows with names above each sequence indicate primers (Table S2) targeting the corresponding positions of the sequences (not to scale). Amplicon sizes were shown in between each pair of primers. (B) Mutant confirmation by PCR. The primers EBGNHe-5/EBGh3-3 and PR1655/PR1656 were used for the pKOBEG-Apra and pFLP2-Apra determinations, respectively. The expected amplicon sizes are given in brackets. (C) Mutations were confirmed via sequencing. Only the coding sequences are shown. Red text indicates the corresponding codons. Download FIG S5, TIF file, 4.4 MB. Copyright © 2023 Hu et al. 2023 Hu et al. https://creativecommons.org/licenses/by/4.0/ This content is distributed under the terms of the Creative Commons Attribution 4.0 International license.

### Quinolones and cephalosporins directly bind to the extracellular sensor domain of PhoQ.

We hypothesized that antibiotics trigger the phosphorylation of PhoQ by binding to the periplasmic sensor domain of PhoQ (PhoQ^SD^). To test this, the truncated PhoQ^SD^ was expressed in E. coli BL21 (Fig. S6), where PhoQ^SD^ was found in a dimer form, according to the native-PAGE (data not shown). Differential scanning calorimetry (DSC) and microscale thermophoresis (MST) assays were performed to evaluate the interactions between the PhoQ^SD^ dimer and antibiotics.

10.1128/mbio.03395-22.7 FIG S6 Expression and purification of His-tagged PhoQ^SD^ in E. coli BL21. (A) Cloning of the *phoQ* sensor domain fragment. M, 100 bp marker. (B) SDS-PAGE and Western blot analysis of bacterial lysate. Lane 1, the BL21 strain with the empty plasmid. Lanes 2 to 4, the BL21 strain with the pET28a-*hisphoQ*^SD^ plasmid. M, protein marker. (C) Purification of His-tagged PhoQ^SD^ protein via Ni-NTA column. Lane 1, bacterial whole protein after sonication. Lanes 2 to 12, different gradients of the imidazole (20mM, 30mM, 50mM, 100mM, and 250mM) flux protein. M, protein marker. Download FIG S6, TIF file, 3.1 MB. Copyright © 2023 Hu et al. 2023 Hu et al. https://creativecommons.org/licenses/by/4.0/ This content is distributed under the terms of the Creative Commons Attribution 4.0 International license.

For the DSC measurements, PhoQ^SD^ was adjusted to 20 μM with or without antibiotics (100 μM) in 10 mM sodium phosphate buffer (pH 7.4 or 5.5). A sharp exothermic peak was detected to conform to the thermal denaturation temperature (*T_m_*) melting curves of PhoQ^SD^, PhoQ^SD^-NAL, and PhoQ^SD^-CAZ at 52.29°C, 51.00°C, and 50.54°C under neutral conditions, respectively (Fig. 5C). Similarly, under slightly acidic conditions (pH 5.5), PhoQ^SD^ melted at a *T_m_* of 55.79°C (Fig. 5D), which was higher than that observed in the presence of NAL or CAZ (53.88°C and 54.27°C, respectively). These data suggested that PhoQ^SD^ could bind to the antibiotics and further destabilize their tertiary structures.

For the MST tests, the recombinant PhoQ^SD^ was titrated with various concentrations of antibiotics, and the raw data of the fluorescence time trace in 16 capillaries are shown in Fig. S7. As a positive control, we measured the binding affinity of colistin with PhoQ^SD^. As the concentration of colistin increased, PhoQ^SD^ displayed an enhanced binding affinity to colistin, which formed a thermophoretic amplitude with a dissociation constant (*K_d_*) value of 6.51 ± 0.35 μM (Fig. 5E) and a well-fitted fluorescence curve. Notably, when the same procedure was applied for NAL, CAZ, or ofloxacin, we also obtained well-fitted curves of fluorescence changes in the thermophoretic amplitude, with *K_d_* values of 24.73 ± 4.30 μM, 25.63 ± 13.42 μM, and 33.08 ± 28.28 μM, respectively. The interactions between PhoQ^SD^ and sulfamethoxazole/azithromycin displayed poor affinity with the dispersed fluorescence. Interestingly, the fluorescence of PhoQ^SD^ binding to CAZ decreased as the antibiotic concentration increased, in contrast to what was observed with antibiotics. This might be attributable to the different binding sites in PhoQ^SD^. These data revealed the specific binding affinity of PhoQ to NAL, CAZ, and ofloxacin.

10.1128/mbio.03395-22.8 FIG S7 The raw microscale thermophoresis results. (A–D) The initial fluorescence and fluorescence time trace in the 16 capillaries as well as the dose response curve of PhoQ^SD^ bound to different concentrations of colistin (A), nalidixic acid (B), ceftazidime (C), and ofloxacin (D). The RED-tris-NTA 2nd fluorescent dye was used to label the His-tagged PhoQ^SD^. The fluorescence time trace represents capillary 1 to capillary 16, in turn. With decreasing antibiotics concentration, the sample in the capillary changed from the bound state (capillary 1) to the unbound state (capillary 16). The red area (cursor positions) was recorded for the calculation of the thermophoretic amplitude. The dose response curve was used to calculate the *K_d_* value. Download FIG S7, TIF file, 1.7 MB. Copyright © 2023 Hu et al. 2023 Hu et al. https://creativecommons.org/licenses/by/4.0/ This content is distributed under the terms of the Creative Commons Attribution 4.0 International license.

### The internal cavity in the PhoQ sensor domain is critical for the recognition of antibiotics.

**FIG 6:**
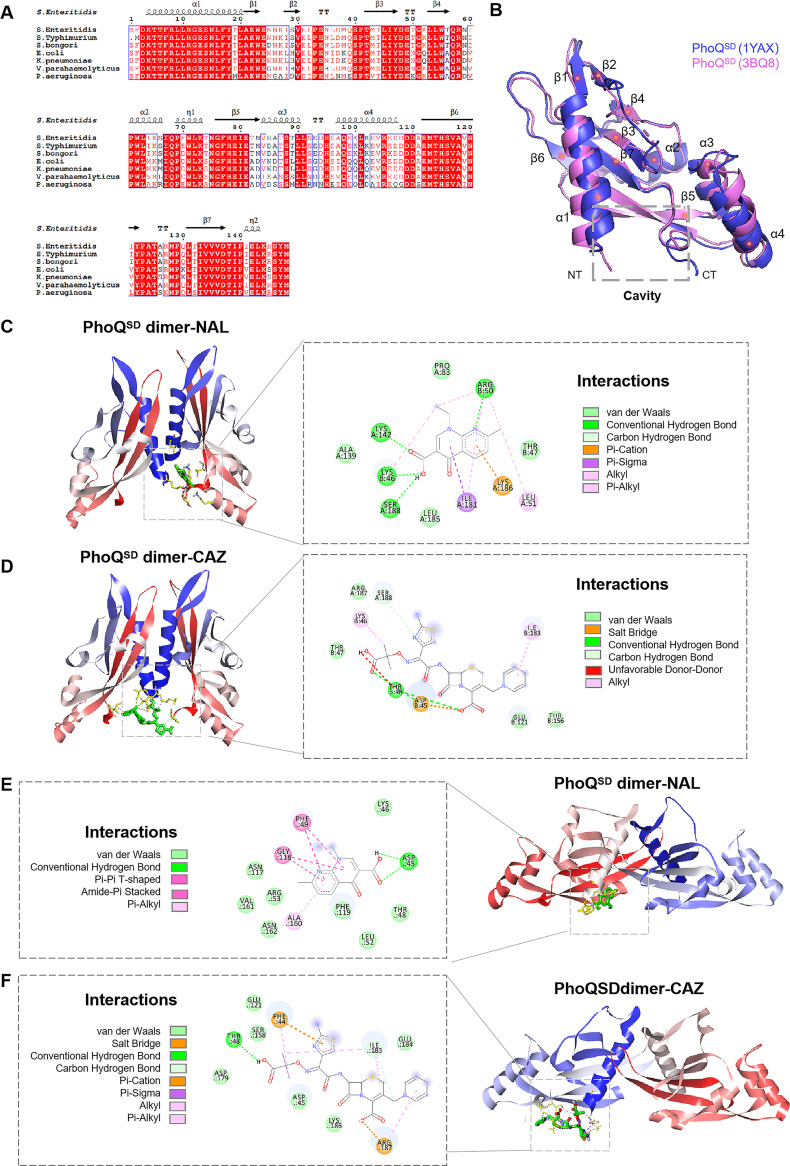
Structures of *S.* Enteritidis PhoQ and molecular docking analysis. (A) Alignment of PhoQ^SD^ sequences from multiple species. Identical regions are colored red, and conserved regions (identity > 70%) are shown in boxes. (B) Homologous modeling of *S.* Enteritidis PhoQ^SD^. The crystal structures of E. coli PhoQ (PBD ID: 3BQ8) and *S.* Typhimurium PhoQ (PBD ID: 1YAX) were used as the templates. NT, N terminus; CT, C terminus. (C and D) Molecular docking results for NAL (C) and CAZ (D) with *S.* Enteritidis PhoQ^SD^ dimer (3BQ8 as the template). (E and F) Molecular docking results for NAL (E) and CAZ (F) with *S.* Enteritidis PhoQ^SD^ dimer (1YAX as the template).

To clarify the spatial position in which the interaction between NAL/CAZ and PhoQ^SD^ occurred, molecular docking was used to model and verify their binding at the atomic level. The evolutionary conservation of the PhoQ sensor was estimated, based on the homologous sequence of phylogeny, using Consurf. The sequences of PhoQ^SD^ (residues 45 to 188) from *S.* Enteritidis, *S.* Typhimurium, S. bongori, E. coli, Klebsiella pneumoniae (K. pneumoniae), Vibrio parahaemolyticus (V. parahaemolyticus), and P. aeruginosa were aligned, suggesting relatively conserved primary and secondary structures of PhoQ among various species (Fig. 6A). *S.* Enteritidis PhoQ^SD^ was composed of a mixed α/β-structure with a central, seven-stranded β sheet core, and it shared an amino acid sequence identity of >81.08% with other species. In addition, the homologous modeling of the three-dimensional structure of *S.* Enteritidis PhoQ^SD^ was carried out using the crystal structures of E. coli and *S.* Typhimurium PhoQ (PDB ID: 3BQ8 and 1YAX) as the templates. The two monomer structures of PhoQ^SD^ were highly conserved and formed a cavity pocket in the N terminus and in the C terminus.

Given that PhoQ was anchored to the intracellular membrane in a dimeric form and that the dimeric crystal structures of the PhoQ sensor domain of E. coli and *S.* Typhimurium show different conformations, we constructed two models of the *S.* Enteritidis PhoQ^SD^ dimer for molecular autodocking. When NAL was docked into the PhoQ^SD^ dimer (using 3BQ8 as the template), seven residues (Lys46, Arg50, Leu51, Lys142, Ile181, Lys186, and Ser188) were found to be located at a distance of within 5 Å to NAL, among which Lys46, Arg50, Lys142, and Ser188 formed hydrogen bonds with the NAL molecule (Fig. 6C). Besides that, CAZ was docked into 8 residues (Phe44, Asp45, Thr48, Ser158, Asp179, Ile183, Lys186, and Arg187) of PhoQ^SD^ by binding to Thr48 and Ser188 via hydrogen bonds (Fig. 6D). In contrast to NAL, which recognized the two N termini and the one C terminus of the PhoQ^SD^ dimer, CAZ was prone to binding in the horizontal line of the two N-terminus and C terminus central positions. These residues were almost located in the cavity pocket of PhoQ^SD^, and this binding model would provide a vacant space facing the cell membrane. The lowest binding energy that was observed in the PhoQ^SD^ dimer with NAL or CAZ was −6.60 kcal/mol or −4.55 kcal/mol, respectively (Table 1). Notably, the crystal structure of *S.* Typhimurium PhoQ^SD^ (PDB ID: 1YAX) was in a Ca^2+^-bound state, and the monomer was in close proximity to the inner membrane, leading to PhoQ repression. Therefore, NAL and CAZ were only bound to the PhoQ^SD^ monomolecular when *S.* Typhimurium PhoQ^SD^ was used as the template, with the lowest binding energies of −6.10 and −4.48 kcal/mol, respectively (Fig. 6E and F) (Table 1).

**TABLE 1:** The results of molecular docking between the PhoQ sensor domain and antibiotics

Template (PDB ID)	Receptor (protein)	Compounds (antibiotics)	Binding energy (kcal/mol)	Key amino acids for interaction	Hydrogen bonds
1YAX	PhoQ^SD^ dimer	CAZ	−4.88	Phe_44_Asp_45_Thr_48_Glu_121_Ser_158_Asp_179_Ile_183_Glu_184_Lys_186_Arg_187_	Thr_48_Ile_183_Arg_187_
		NAL	−6.10	Asp_45_Lys_46_Thr_48_Phe_49_Leu_52_Arg_53_Asn_117_Gly_118_Phe_119_Ala_160_Val_161_Asn_162_	Asp_45_
3BQ8	PhoQ^SD^ dimer	CAZ	−4.55	Asp_45_Lys_46_Thr_47_Thr_48_Glu_121_Thr_156_Ile_183_Arg_187_Ser_188_^*a*^	Thr_48_Ser_188_^*a*^
		NAL	−6.60	Lys_46_Thr_47_Arg_50_Leu_51_Pro_83_Ala_139_Lys_142_Ile_181_Leu_185_Lys_186_Ser_188_^*a*^	Lys_46_Arg_50_Lys_142_Ser_188_^*a*^

a The amino acids that are unmarked are located in the A chain of the PhoQ^SD^ dimer. The amino acids that are underlined were located in the B chain of the PhoQ^SD^ dimer. NAL, nalidixic acid; CAZ, ceftazidime.

**FIG 7:**
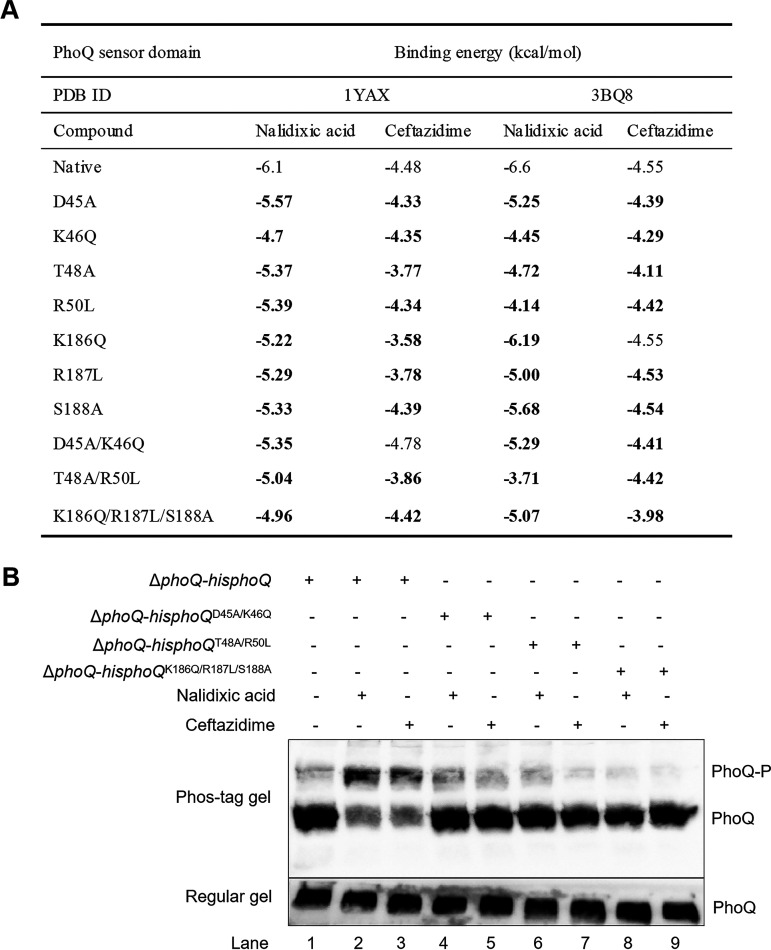
The mutagenesis of the residues surrounding the cavity abrogated PhoQ phosphorylation. (A) The binding energy values of NAL and CAZ with native and variant PhoQ^SD^. The entries in bold typeface indicate the PhoQ^SD^ variants with higher binding affinity to NAL/CAZ than that of the native PhoQ^SD^; (B) Phosphorylation assays of the native PhoQ and its variants (D45A, K46Q, T48A, R50L, K186Q, R187L, and S188A) in the indicated strains.

The virtual mutagenesis of the screened amino acids located in the PhoQ^SD^ cavity, including 5 basic residues (Lys46, Thr48, Arg50, Arg187, and Lys186), an acidic residue (Asp45), and a polar residue (Ser188) was performed. The docking results suggested that the PhoQ^SD^ variants with each mutation displayed enhanced binding energy with antibiotics, particularly those with double and triple mutations (Fig. 7A). Furthermore, these residues were replaced with small, nonpolar residues of alanine, glutamine, or leucine to obtain the Δ*phoQ*-*hisphoQ*^mutant^ strains. All of the strains were cultured in LB with NAL/CAZ, and the cell lysates were obtained for a phosphorylation analysis. A shifted band of PhoQ was observed in the Δ*phoQ*-*hisphoQ* cell lysate, whereas the intensity of such a shifted band almost disappeared in the variants of Δ*phoQ*-*hisphoQ*^D45A/K46Q^, Δ*phoQ*-*hisphoQ*^T48A/R50L^, and Δ*phoQ*-*hisphoQ*^K186Q/R187L/S188A^ (Fig. 7B, lanes 3 to 9), strongly suggesting that these residues were crucial for PhoQ autophosphorylation. Therefore, the PhoQ cavity was crucial for the recognition of antibiotics, which allowed for PhoQ autophosphorylation and signal transduction to PhoP.

## DISCUSSION

Gram-negative bacterial cells are encased by a trilaminar envelope that is constituted by a thin peptidoglycan layer that is sandwiched between two membranes (the outer membrane and the inner membrane). This structure serves as a mechanistic barrier that defends against antibiotics of various actions. Typically, hydrophobic quinolones reach their intracellular targets by diffusion through the lipid bilayer of the outer membrane, whereas hydrophilic cephalosporins penetrate the outer membrane through porins or selective channels (44). This prompted us to wonder whether the PhoPQ-mediated resistance of *S.* Enteritidis to these antibiotics was achieved via the rearrangement of envelope entities so as to control the intracellular accumulation of antibiotics. The RNA-seq results revealed that the envelope-associated events were largely affected by PhoPQ, including the modification of the LPS surface charge, the alteration of the fatty acid composition, and the interruption of peptidoglycan synthesis.

The phenotypic observations caused by the loss of PhoP supported the RNA-seq results and indicated that cells lacking the PhoP regulator, when exposed to quinolones and cephalosporins, underwent envelope perturbation that was favorable to the killing of antibiotics. A prominent example was the expressional alteration of the LPS modification genes, which led to an unstable membrane with a smaller amount of negatively charged LPS, which was prone to an influx of massive hydrophobic NAL or hydrophilic CAZ. Previous studies have also demonstrated the contribution of PhoPQ to LPS homeostasis in *S.* Typhimurium and E. coli, where PhoPQ modulates the remodeling of the lipid A domain of LPS in the presence of CAMPs or low concentrations of Mg^2+^ (25, 45). The observed upregulation of the outer membrane porin genes (Fig. 2D), combined with the nature of porins to promote the entry of antibiotics, could, to some extent, explain the increased sensitivity of the cells lacking PhoP. In addition, cells with membranes of high fluidity are vulnerable to environmental stress (46). Given the contribution of UFAs to membrane fluidity, the higher level of expression in UFAs (Fig. 3C) might also explain the envelope perturbation in the Δ*phoP* cells. Moreover, the peptidoglycan layer is another critical barrier that undertakes the guard of cells, with a meshwork formed by cross-linked glycan strands (38, 39, 47). The downregulated genes related to peptidoglycan biosynthesis and cross-linking in Δ*phoP* cells (Fig. 2C) suggested that PhoPQ acts to augment peptidoglycan synthesis to compensate for the loss due to cell wall hydrolysis by CAZ. These data collectively revealed a role of PhoPQ in the coordination of envelope components to limit the intracellular accumulation of antibiotics, which might be also available against other antibiotics, as envelope-mediated resistance is not specific to one or several classes of antibiotics.

**FIG 8:**
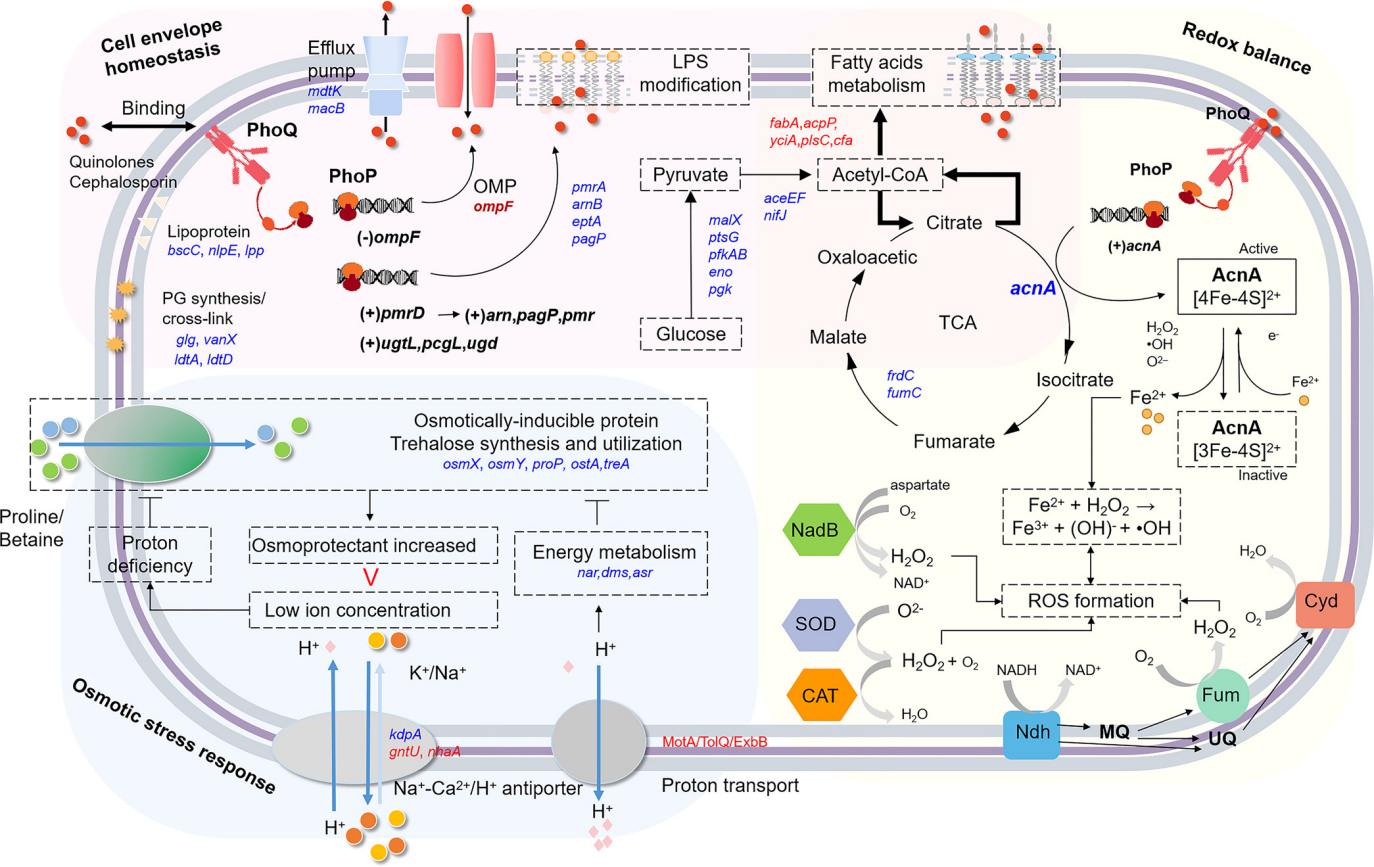
Proposed model for the regulation of envelope homeostasis, the osmotic stress response, and the redox balance by PhoPQ. (A) The upregulated and downregulated genes in Δ*phoP*, versus those of te hWT, are marked in red and blue, respectively.

Osmolarity is known as one of the host environmental factors to bacteria, and PhoPQ perceives an osmotic upshift and induces the activation of virulence factors (23). Consistently, our RNA-seq results revealed that the absence of PhoP did reduce the expression of *pagN* as well as the SPI-2 type III secretion system-related operons (*ssa*, *ssc*, and *sse*) (Fig. S2), which are the major factors that are responsible for the Salmonella adhesion and invasion of host cells (48–50). These findings might provide the basis for an explanation of the synchronized occurrence of resistance and pathogenicity. The reason for a hyperosmotic response in a nonhyperosmotic environment (LB broth) in this study might be explained by the proton-leaving. Proton antiporters are responsible for export of cations, which leads to a decreased cytoplasmic osmotic pressure, but this descending osmotic pressure could be offset by the osmoprotectant transport system, which was functional with cotransport protons and required energy to be supplied (51, 52). In this work, for the first time, we observed the upregulation of the MotA/TolQ/ExbB proton channel family genes and the downregulation of the osmotic stress-related genes in Δ*phoP* cells (Fig. 2E). In addition, in Δ*phoP* cells, the expression of energy-producing systems, operons, and genes (*dms*, *cyd*, *asr*, *pta*, *frdC*, etc.) was also dramatically downregulated (Fig. S2C). Therefore, we assumed that the deficiency in PhoPQ causes proton-leaving and an osmotic pressure imbalance under the pressure of antibiotics (Fig. 8), and this ultimately retarded cell viability under NAL and CAZ.

The production of ROS, including H_2_O_2_, the hydroxyl radical (•OH), and the superoxide anion radical (O_2_^–^), is among the bactericidal pathways that are shared by drugs of various actions (53, 54). Our findings suggested that NAL treatments and CAZ treatments remarkably increased ROS production and decreased the NAD^+^/NADH ratio in Δ*phoP* cells. Our findings also demonstrated the transcription of the genes that are responsible for ROS formation and iron transport as well as the decreased transcription of the genes that are responsible for ROS scavenging, Fe-S cluster assembly, and the NADH-related electron transport chain (55–62), which resulted in an ROS-generating Fenton reaction and an intracellular redox imbalance (Fig. 8). On the other hand, it was reported that the antibiotic treatments could stimulate ROS production by hyperactivating the bacterial central metabolism (e.g., the tricarboxylic acid cycle) (63, 64). The downregulated expression of the tricarboxylic acid cycle-related genes *acnA*, *fumC*, and *frdC* in Δ*phoP* cells (Fig. S2C) suggested a positive regulation of PhoPQ in the central metabolism. These data indicated that the regulatory pathways of PhoPQ were complex but closely linked so that they could sustain a rigid envelope and cytoplasm homeostasis.

The RNA-seq data, combined with phenotypic validations, allowed us to understand which downstream genes are influenced by the PhoP regulator. Based on the PhoP box motif sequence, we further discriminated 68 putative gene candidates that were directly targeted by PhoP from all of the DEGs in the Δ*phoP* cells, of which the negative regulon *ompF* and the positive regulon *acnA* were identified for the first time via EMSA and *lacZ* reporter assays. These direct targets might reflect the emergency measurements that are immediately taken by *S.* Enteritidis once stressed by antibiotics. The negatively regulated *ompF* encodes an outer membrane porin that prohibits the diffusion and entry of a variety of small molecules, including quinolones and β-lactams, into the cell. The positively regulated *acnA* encodes a citric acid cycle enzyme aconitase that responds to the superoxide anion and hydrogen peroxide to facilitate energy production and prevent the liberation of reactive iron from oxidative damage (65). This implies that the reduced expression of OM porins as well as the maintenance of intracellular basic metabolism and the redox balance might serve as the “first aid” in *S.* Enteritidis when it is confronted with antibiotics.

Having gained information about how PhoP regulates the genes to deal with the stresses from antibiotics, we explored how this TCS exploits the “sentry” PhoQ to report their presence to PhoP. Although TCSs are widely distributed across bacterial species, there are limited details regarding the signal transduction and the intact crystal structure for histidine kinases (66–68). The PhoQ periplasmic sensor domain was reported to respond to low divalent cations (21, 69), an acidic pH (22), CAMPs, and the small regulator protein SafA (70, 71). Here, we surprisingly found that quinolones and cephalosporins could directly interact with the cavity of PhoQ sensor domain and that this binding mode provided a vacant space for conformational changes of PhoQ for activity control and autophosphorylation. It was previously shown that SafA directly interacts with the cavity structure of PhoQ for activation, and the mutagenesis of the residues surrounding the cavity attenuated the SafA-mediated activation of PhoQ (70, 71). In this study, the mutagenesis of the residues (D45, K46, T48, R50L, K186, R187, and S188) in the cavity of PhoQ additionally enhanced its binding energy with antibiotics and abrogated its phosphorylation, suggesting that the antibiotics of quinolone and cephalosporin could activate PhoQ in a similar way as SafA. Therefore, we concluded that this binding mode enables the activation of PhoQ via a conformational change and the delivery of a signal to PhoP. Altogether, *S.* Enteritidis PhoQ directly senses quinolones or cephalosporins to activate PhoPQ regulation in a rapid way without damage to the integrity of the cell envelope, which may represent a novel mechanism by which to defend against quinolones or cephalosporins.

Conclusively, we have demonstrated the transcriptional regulation of PhoPQ on bacterial envelope integrity, the osmotic stress response, and the redox balance, which enabled resistance to quinolones and cephalosporins in *S.* Enteritidis. The histidine kinase PhoQ is an antibiotic sensor that initiates signal transduction, leading to a phosphorylated PhoP that activates or represses the transcription of target genes (*ompF* and *acnA*) in response to antibiotics. Therefore, we have proposed a schematic model illustrating how the membrane-associated histidine kinase PhoQ signals the presence of antibiotics and dispatches PhoP to trigger the expression of overall genes to adapt to antibiotic-caused stress (Fig. 8). In future work, it would be feasible to develop PhoPQ inhibitors, based on the novel target genes identified in our work, to potentiate the efficacy of quinolones or cephalosporins in treating infections caused by *S.* Enteritidis, which may provide a promising means by which to inhibit antibiotic resistance.

## MATERIALS AND METHODS

### Strains and culture conditions.

*S.* Enteritidis SJTUF12367 (GenBank assembly accession no. GCA_004323915.1; https://www.ncbi.nlm.nih.gov/assembly/GCF_004323915.1/) and its derivatives were used in this study (35). The complete list of bacterial strains, vectors, and primers was shown in Table S1. Bacterial routine culture was performed in lysogeny broth (LB) medium at 37°C. Unless otherwise specified, the subinhibitory concentration antibiotic of 32 μg/mL nalidixic acid or 2 μg/mL ceftazidime was added to the medium treatment for 8 h. All strains could grow, and the Δ*phoP* mutant showed a susceptible phenotype.

10.1128/mbio.03395-22.9 TABLE S1 The strains, plasmids, and primers used in this study. Download Table S1, DOCX file, 0.03 MB. Copyright © 2023 Hu et al. 2023 Hu et al. https://creativecommons.org/licenses/by/4.0/ This content is distributed under the terms of the Creative Commons Attribution 4.0 International license.

### RNA-seq and RT-qPCR.

Bacterial cultures in the exponential or log phase in LB were inoculated to antibiotics and taken for RNA extraction at 8 h. The total RNA was extracted using the TRIzol reagent (Invitrogen), referencing the method described by Huang et al. (72). The strand-specific transcriptome library construction and Illumina RNA-seq were performed by Majorbio (Shanghai, China). Three independent, biologically repeated experiments were performed. The integrity of the RNA was confirmed via electrophoresis (Fig. S1A). The transcripts per kilobase per million mapped reads incorporate normalization steps to ensure that the expression levels for different genes and transcripts can be compared across runs. The DESeq2 method was used to calculate the DEGs. Genes with a fold change of ≥2 and a Bonferroni-corrected *P* value (*P*_adj_) of <0.05 in at least one sample were determined to be DEGs. COG and KEGG annotation analyses of the DEGs were carried out with the free, online Majorbio Cloud Platform. The RT-qPCR was run in triplicate and amplified using the TB green Premix *Ex Taq* II reagent (TaKaRa), and the amplification efficiencies of the oligonucleotide primers were listed in Table S2. The relative gene transcriptional levels were determined using the 2^-ΔΔCT^ threshold cycle (CT) method and were converted into log_2_ values (72). The expression of *16S rDNA* was used as an internal reference.

10.1128/mbio.03395-22.10 TABLE S2 Amplification efficiencies of the oligonucleotide primers used for RT-qPCR. Download Table S2, DOCX file, 0.01 MB. Copyright © 2023 Hu et al. 2023 Hu et al. https://creativecommons.org/licenses/by/4.0/ This content is distributed under the terms of the Creative Commons Attribution 4.0 International license.

### Data analysis.

The data analysis was conducted using a two-way analysis of variance (ANOVA) and the Holm-Sidak multiple-comparison test via the Prism8.0.1 program.

### Data availability.

The Gene expression data have been deposited into the NCBI Gene Expression Omnibus (GEO) under the accession number GSE217589. The detailed protocols for the phenotypic analysis, bacterial genetic manipulations, EMSA, DSC, MST, molecular docking, and *in vitro* and *in vivo* phosphorylation assays are provided in the “Text S1” file.

10.1128/mbio.03395-22.1 TEXT S1 Supplemental Methods. 1. Download Text S1, DOC file, 0.08 MB. Copyright © 2023 Hu et al. 2023 Hu et al. https://creativecommons.org/licenses/by/4.0/ This content is distributed under the terms of the Creative Commons Attribution 4.0 International license.
