# Supplementary figures and images for: PhoPQ Regulates Quinolone and Cephalosporin Resistance Formation in Salmonella Enteritidis at the Transcriptional Level
Source: mBio. 2023 May 15;14(3):e03395-22. doi: 10.1128/mbio.03395-22 (PMC10294627; doi:10.1128/mbio.03395-22)

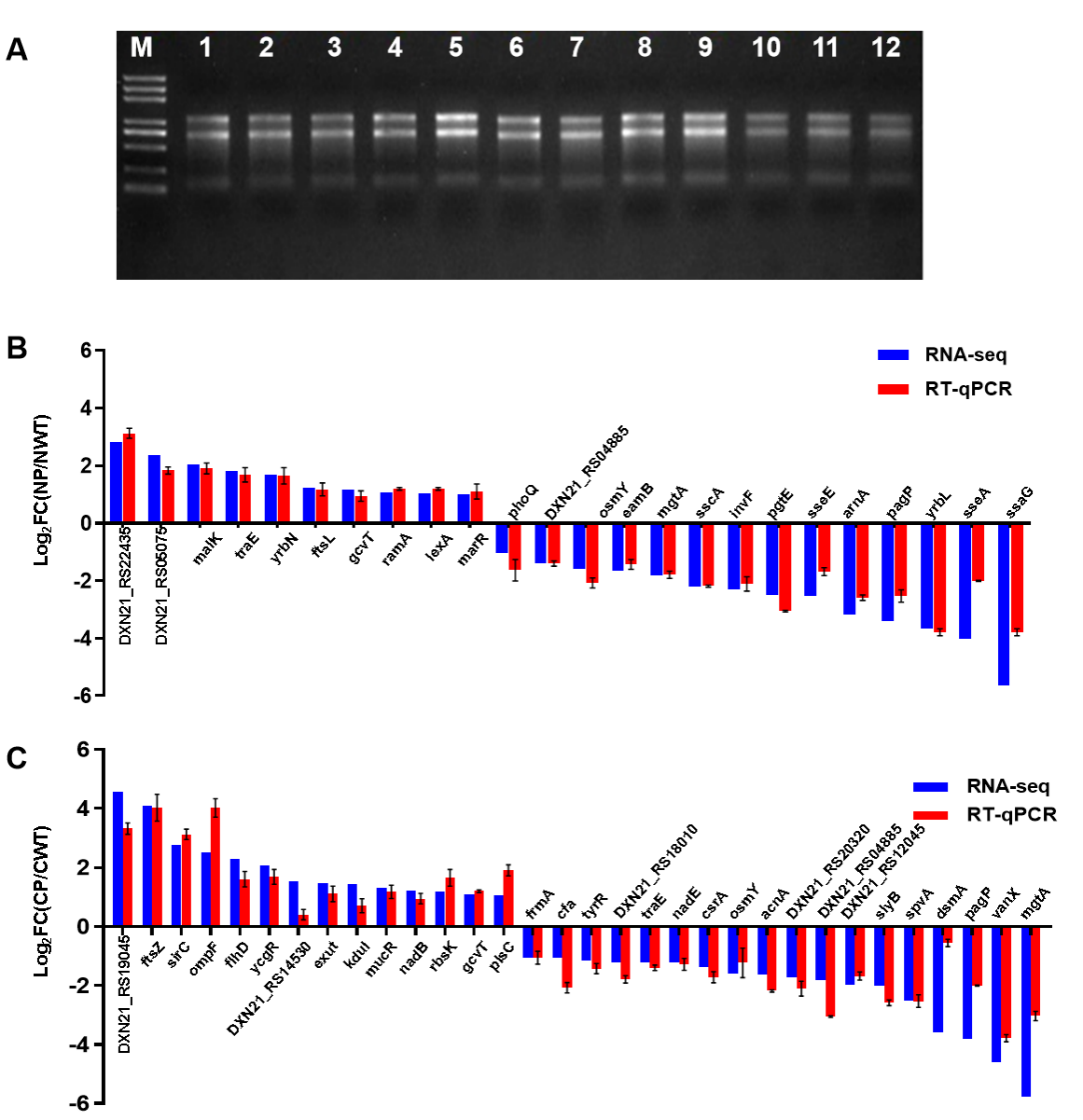

Supplement: FIG S1 [file mbio.03395-22-s0002.tif]

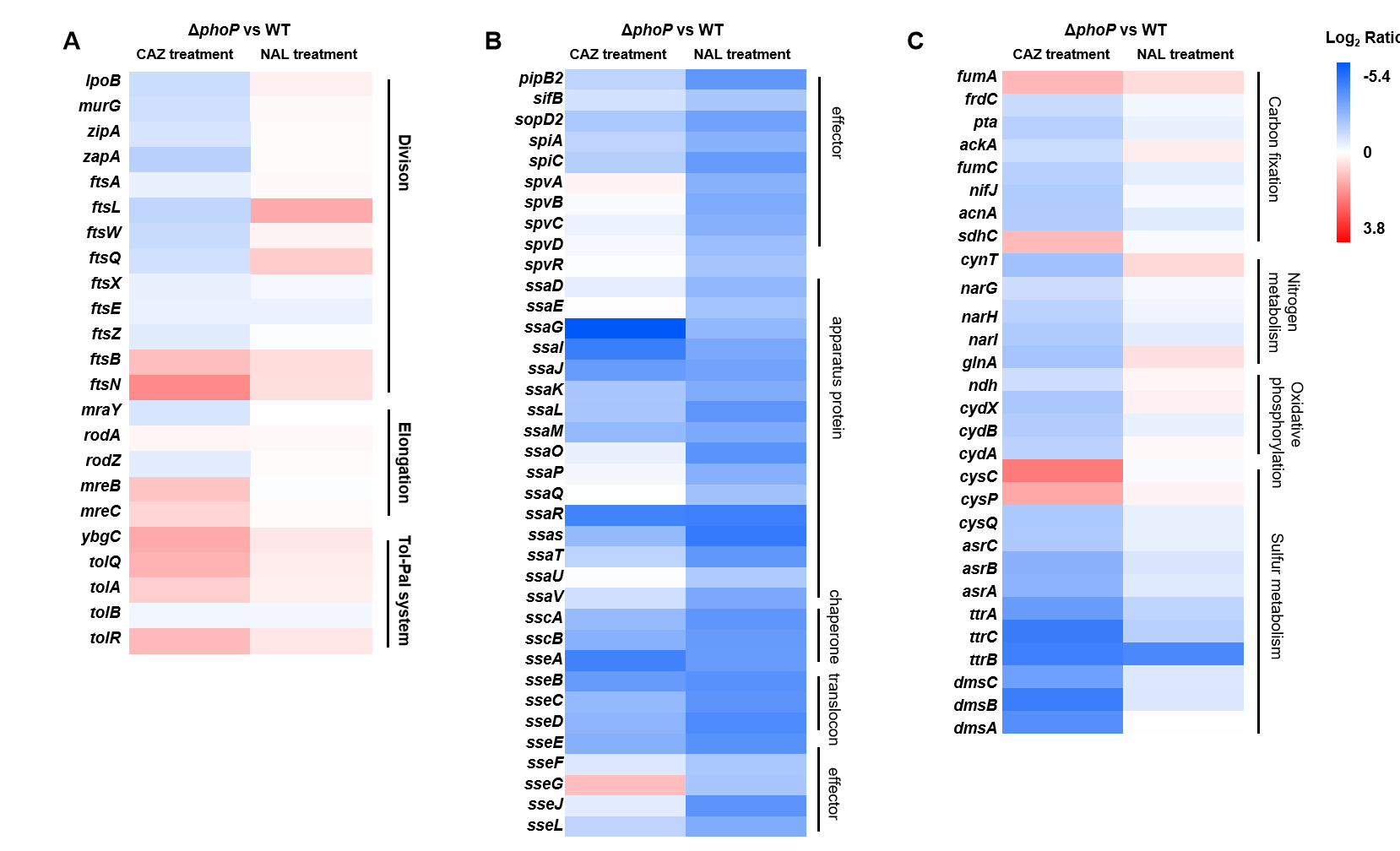

Supplement: FIG S2 [file mbio.03395-22-s0003.tif]

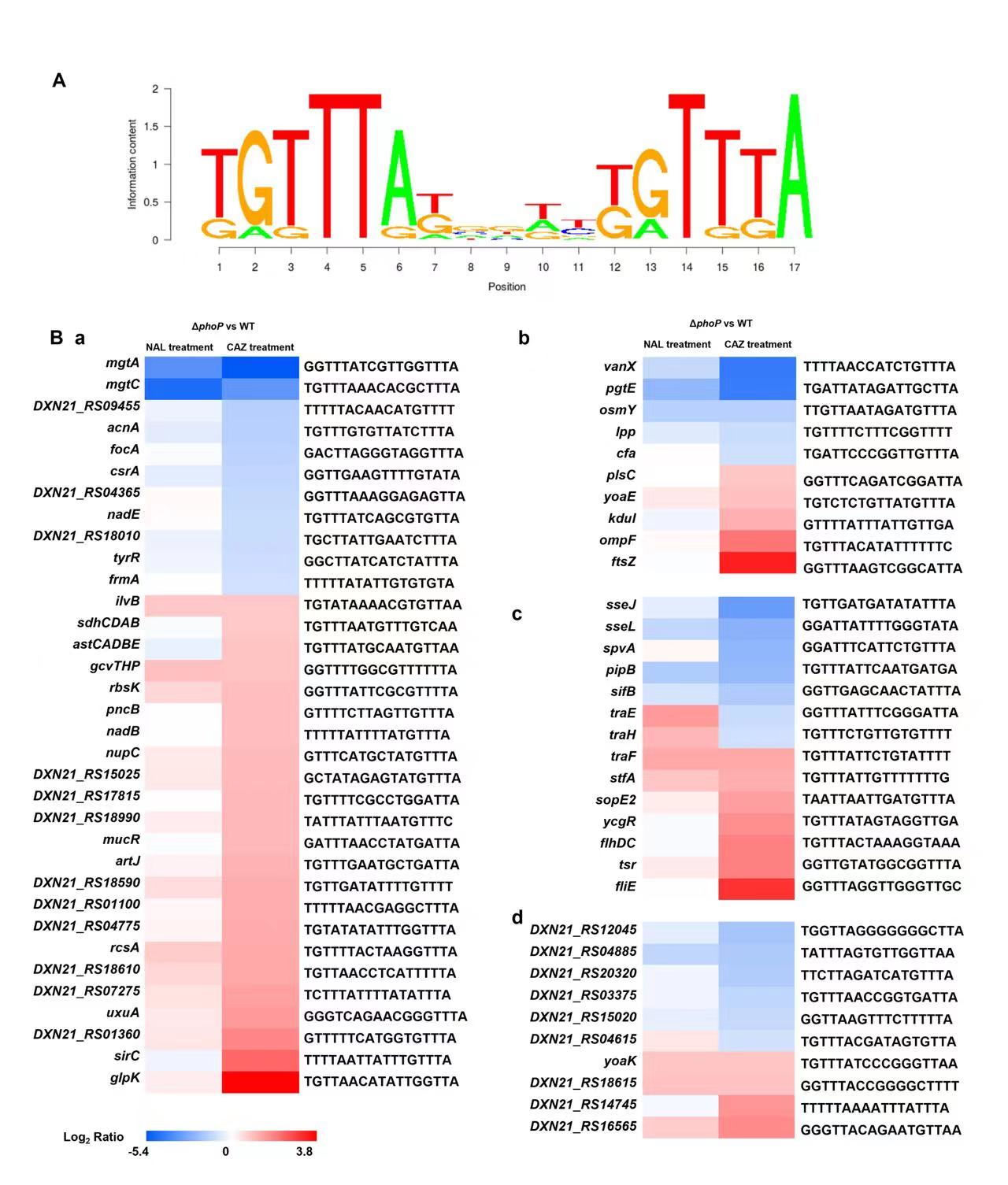

Supplement: FIG S3 [file mbio.03395-22-s0004.tif]

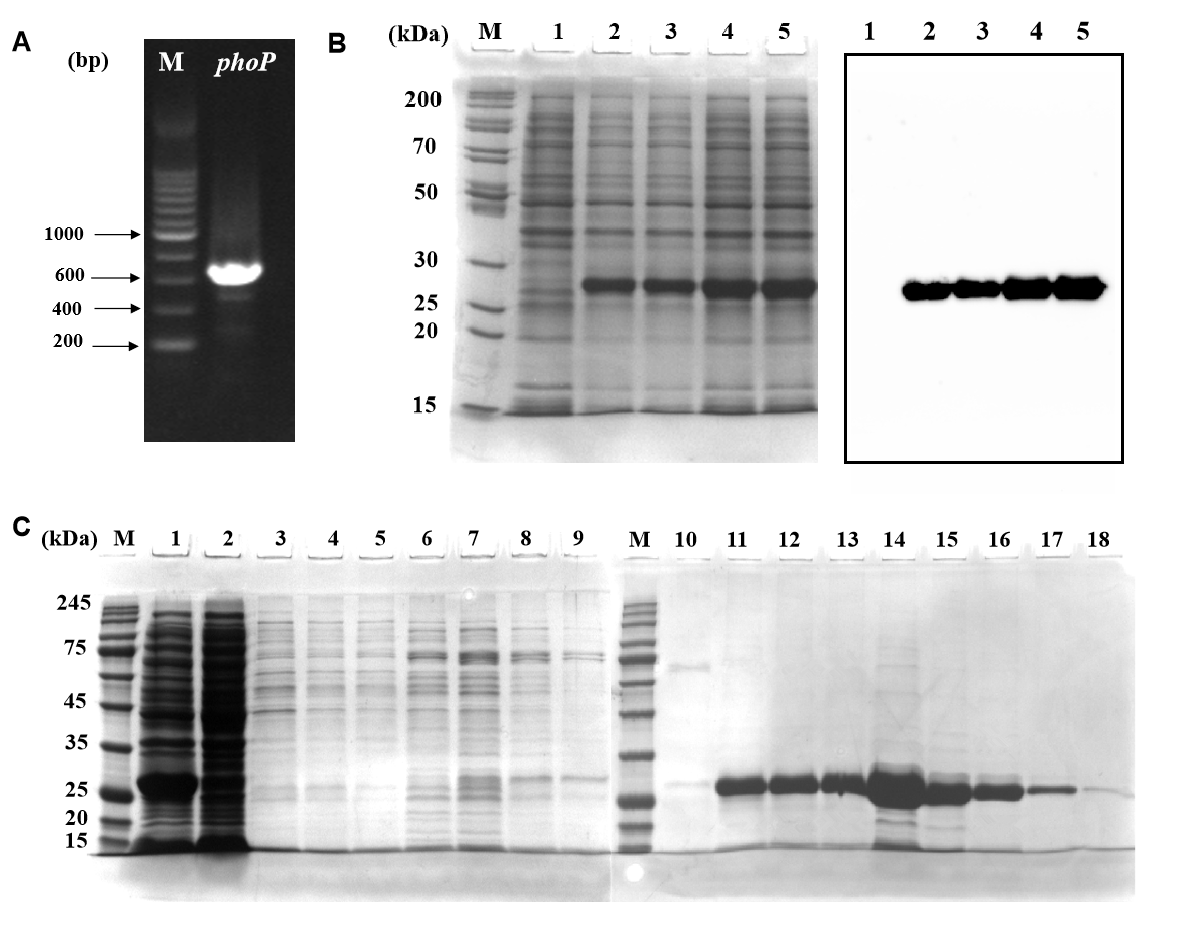

Supplement: FIG S4 [file mbio.03395-22-s0005.tif]

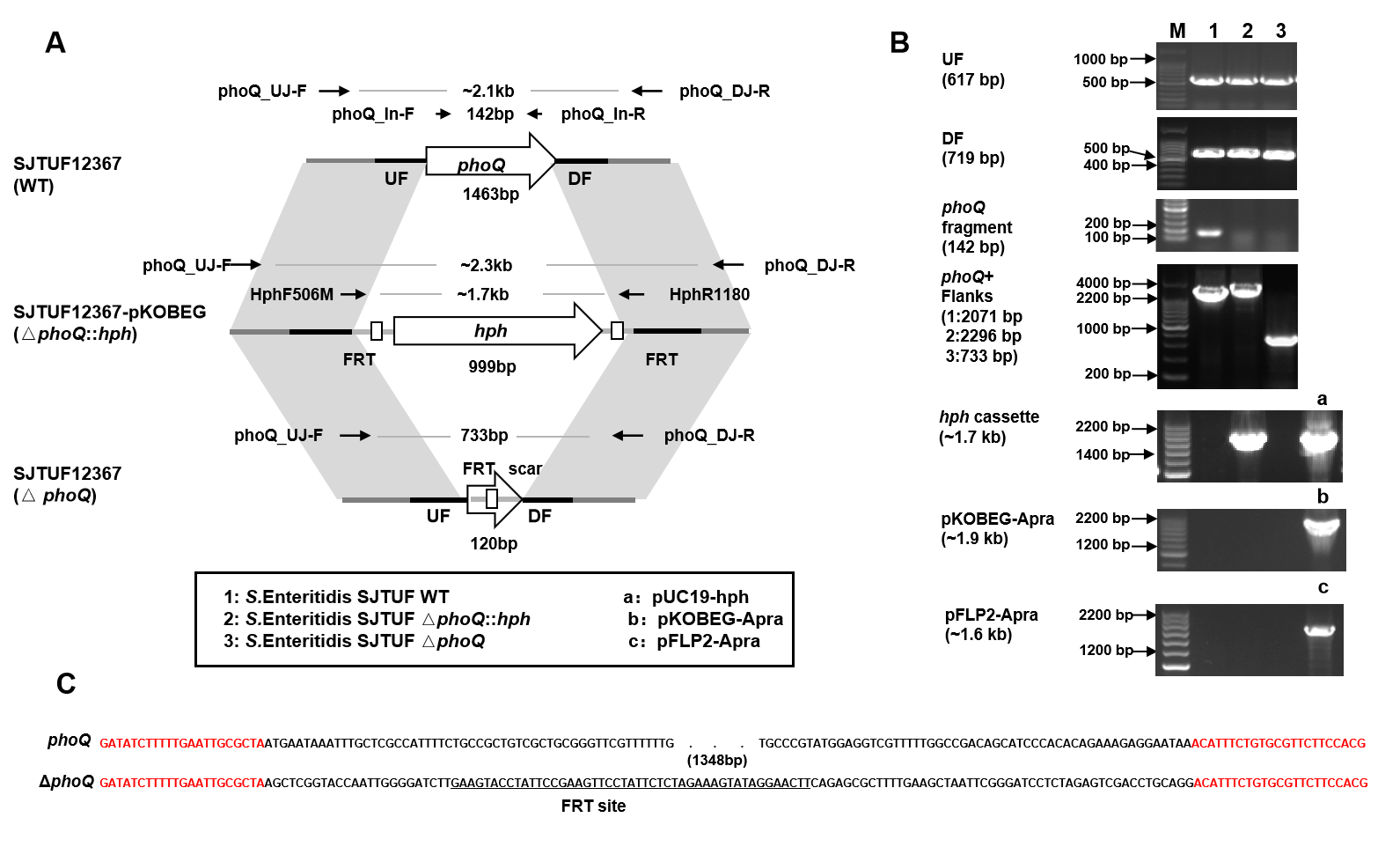

Supplement: FIG S5 [file mbio.03395-22-s0006.tif]

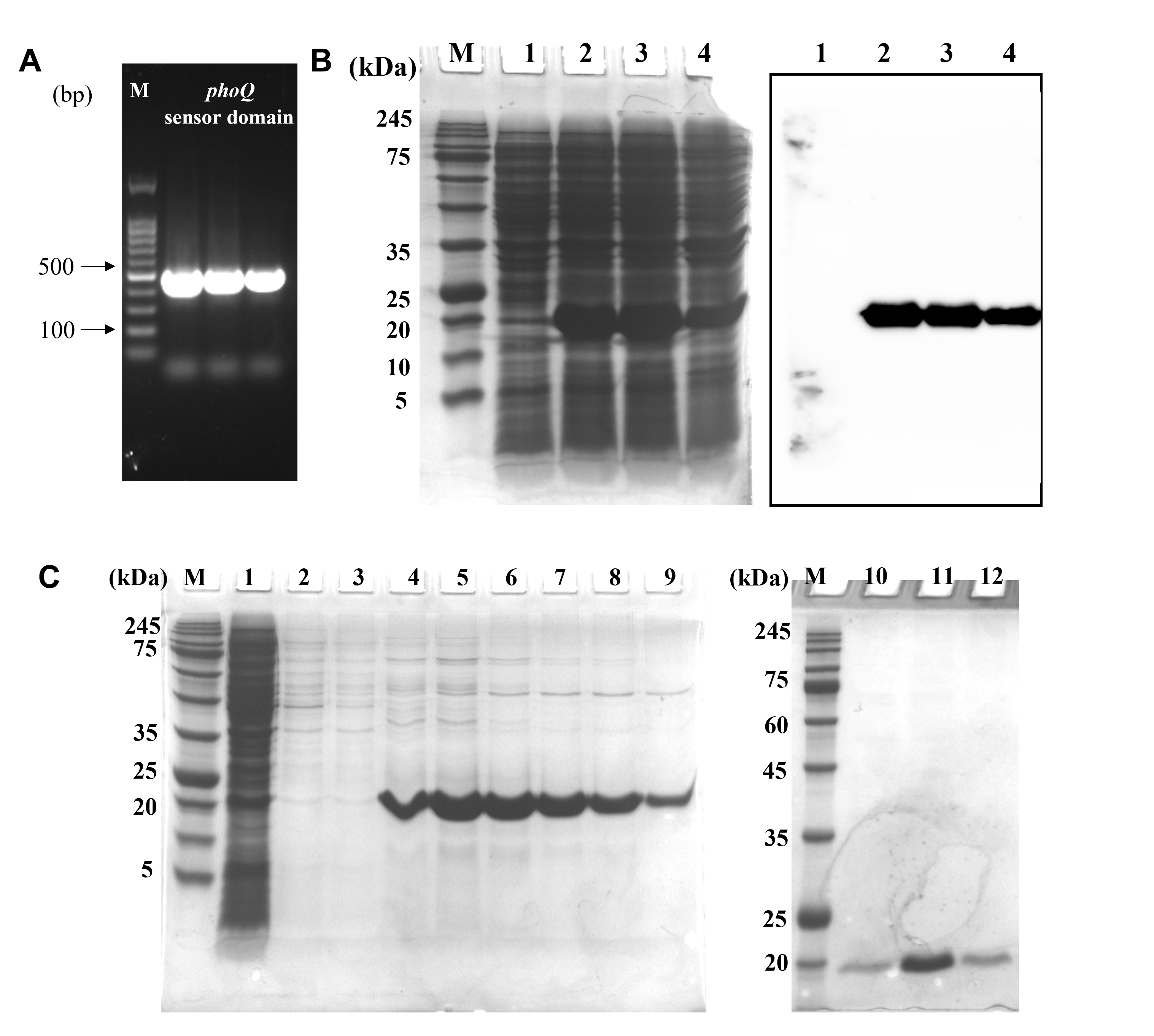

Supplement: FIG S6 [file mbio.03395-22-s0007.tif]

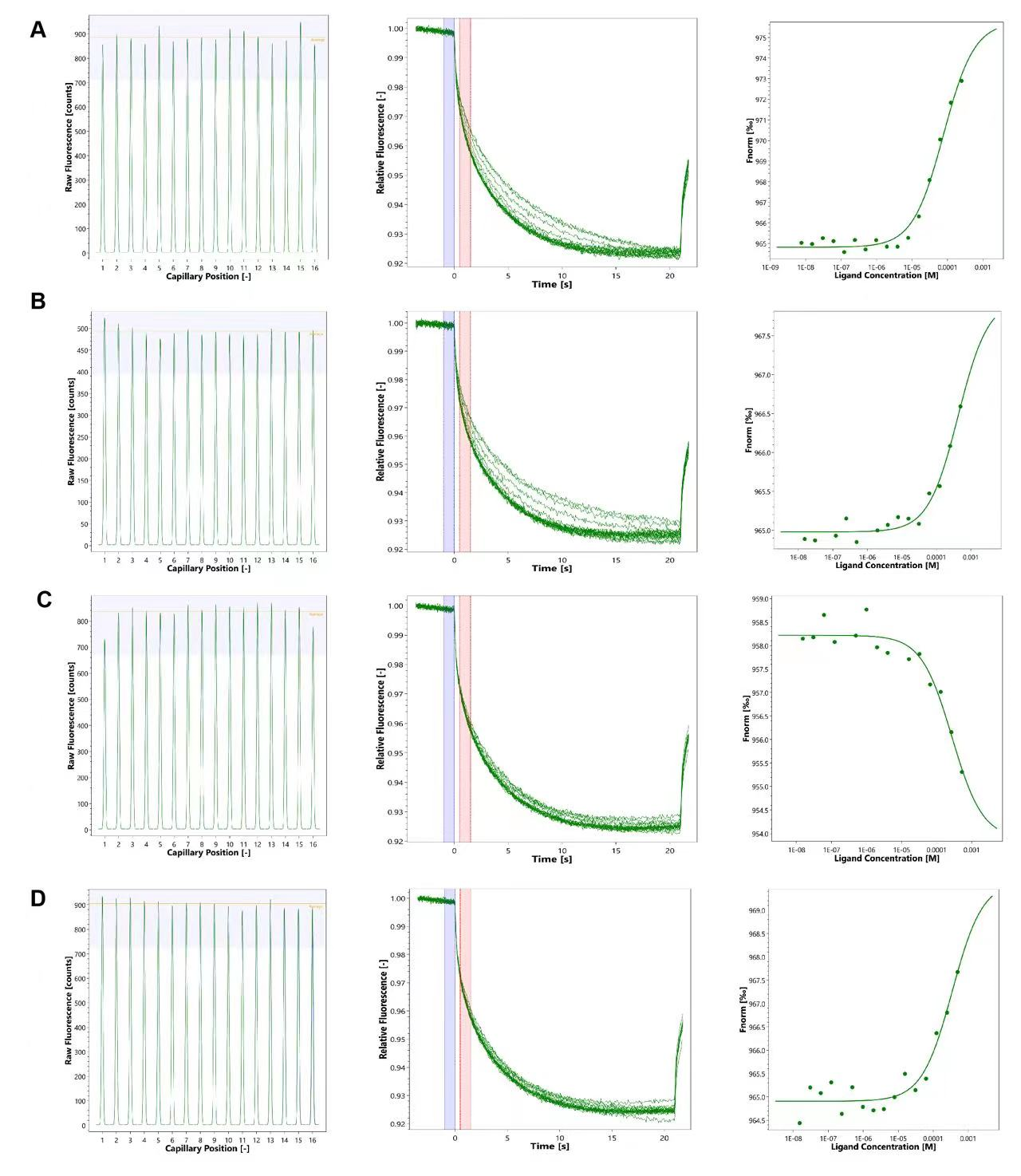

Supplement: FIG S7 [file mbio.03395-22-s0008.tif]
