# Supplementary material for: PhoPQ Regulates Quinolone and Cephalosporin Resistance Formation in Salmonella Enteritidis at the Transcriptional Level
Source: mBio. 2023 May 15;14(3):e03395-22. doi: 10.1128/mbio.03395-22 (PMC10294627; doi:10.1128/mbio.03395-22)
Supplement: TABLE S1 [file mbio.03395-22-s0009.docx]

| Strains and Plasmids | Relevant genotype/description/Sequence (5’-3’) | Source/Target |
| --- | --- | --- |
| Strains | | |
| *S*. Enteritidis SJTUF12367 | multiple drug resistant *S*. Enteritidis, clinical isolate | 1 |
| Δ*phoP* | *phoP* gene deletion *S*. Enteritidis | 2 |
| Δ*phoQ* | *phoQ* gene deletion *S*. Enteritidis | This work |
| Δ*phoQ*-*hisphoQ* | *phoQ* gene complement *S*. Enteritidis |  |
| Δ*phoQ*-*hisphoQ*^D45A/K46Q^ | PhoQ amino acid mutation *S*. Enteritidis |  |
| Δ*phoQ*-*hisphoQ*^T48A/R50L^ |  |  |
| Δ*phoQ*-*hisphoQ*^K186Q/R187L/S188A^ |  |  |
| *E.coli* DH5α | Used for plasmid cloning and galactosidase activity detection | Sangon, China |
| *E.coli* BL21(DE3) | T7 expression |  |
| Plasmids | | |
| pKOBEG-Apra | The plasmid is thermo-sensitive and apramycin-resistant, and contains arabinose-inducible lambda red genes *gam*, *bet* and *exo*. This is a helper plasmid for mutagenesis. | 3 |
| pUC19-Hyg | The plasmid is derived from pUC19. It contains a flippase recognition target (FRT)-flanked *hph* cassette. |  |
| pFLP2-Apra | The plasmid is derived from pFLP2 with the *bla* replaced by an apramycin-resistant gene and was used to flip out the marker gene used for mutagenesis. |  |
| pBAD33-Apra | The plasmid is derived from pBAD33 with the *cat* replaced by an apramycin-resistant gene. It contains an arabinose-inducible vector and was used to compensate gene expression. |  |
| pBAD33-*phoP* | The *phoP* fragment was fused in *EcoR* I and *Xba* I sites, pBAD33 as plasmid skeleton | 2 |
| pBAD33-*hisphoQ* | The His-tag added *phoQ* fragment was fused in *EcoR* I and *Xba* I sites, pBAD33 as plasmid skeleton | This work |
| pBAD33-*hisphoQ*^mutant^ | The His-tag added *phoQ* mutant fragment was fused in *EcoR* I and *Xba* I sites, pBAD33 as plasmid skeleton |  |
| pET-28a | Protein expression vector containing His-tag |  |
| pET28a-*hisphoP* | The N-terminal fusion His-tag expression vector of PhoP protein was constructed from *Nde* I and *Xho* I sites. |  |
| pET28a-*hisphoQ*^SD^ | The C-terminal fusion His-tag expression vector of PhoQ sensor domain protein was constructed from *Nco* I and *Xho* I sites |  |
| pLACZ | The β-galactosidase gene *lacZ* expression vector. | 4 |
| pLACZ-P*_phoP_* | The *lacZ* gene fused with *phoP*/*ompF*/*acnA* promoters using *Nco* I and *Bgl* II sites, pLACZ as plasmid skeleton. | This work |
| pLACZ-P*_ompF_* |  |  |
| pLACZ-P*_acnA_* |  |  |

| Primer | | |
| --- | --- | --- |
| HphF560M | agctcggtaccaattgggga | pUC19-Hyg: *hph* cassette:1686 bp |
| HphR1180 | cctgcaggtcgactctagaggat |  |
| EBGNHe-5 | cccgctagcgaaaagatgtttcgtgaagc | pKOBEG-Apra ~1900 bp |
| EBGh3-3 | gggaagcttattatcgtgaggatgcgtca |  |
| PR1655 | tgctctagagcacggcattttcttttgcgttt | pFLP2-Apra  ~1600 bp |
| PR1656 | cgcggatccgcgtctttaggcccgtagtctgc |  |
| phoQ_UF-F | ccgcagaagatgccagg | Upstream flank of *phoP* UF:617 bp. *hph* cassette overlapped fragment |
| phoQ_UF-R | tacttcaagatccccaattggtaccgagctttagcgcaattcaaaaagat |  |
| phoQ_DF-F | attcgggatcctctagagtcgacctgcaggtgggcgcgcgaaaacc | Downstream flank of *phoP* DF:719 bp *hph* cassette overlapped fragment |
| phoQ_DF-R | gcgaaaaccgaccgaatagt |  |
| phoQ_UJ-F | atcccgccgttccagg | UF*+phoP+*DF gene fragment: 2071 bp |
| phoQ_DJ-R | cgaccaattgctctcaccca |  |
| phoQ_In-F | gaaacgggcaaattattatgg | The fragment of *phoP*:142 bp |
| phoQ_In-R | gaatggtcctcgctcaaca |  |
| hphoQ-F | ccggaattctaacacaagggagaagagatgaataaatttgctcgccatttt | *phoQ* complement fragment：1517 bp, |
| hphoQ-R | gctctagattagtgatggtgatggtgatgttcctctttctgtgtgggat |  |
| pBAD-F | atgccatagcatttttatcc | pBAD33-Apra:205 bp |
| pBAD-R | gatttaatctgtatcagg |  |
| PhoQ^SD^-F | catgccatggccagttttgataaaaccacctttcgt | *phoQ* sensor domain with *Nco* Ⅰ and *Xho I* sites: 453bp, his-tag |
| PhoQ^SD^-R | ccgctcgagttagtgatggtgatggtgatggctccacaccatataggagcgt |  |
| PhoP-F | ggaattccatatgatgcgcgtactggttg | *phoP* with *Nde* I and *Xho* I sites: 694 bp |
| PhoP-R | ccgctcgagttagcgcaattcaaaaagata |  |
| D_45_K_46_ -F | cggctatagcgtaagttttgctcaaaccacctttcgtttgctg | D_45_K_46_ mutated to A_45_Q_46_, the mutant base colored in blue |
| D_45_K_46_ -R | cagcaaacgaaaggtggtttgagcaaaacttacgctatagccg |  |
| T_48_R_50_ -F | gtaagttttgataaaaccgcctttcttttgctgcgcggcga | T_48_R_50_ mutated to A_48_L_50_ |
| T_48_R_50_ -R | tcgccgcgcagcaaaagaaaggcggttttatcaaaacttac |  |
| K_186_R_187_S_188_-F | ccattccgatagaactacaactcgcctatatggtgtggagctggt | K_186_R_187_S_188_ mutated to Q_186_L_187_A_188_ |
| K_186_R_187_S_188_-R | accagctccacaccatataggcgagttgtagttctatcggaatgg |  |
| acnA(FAM)-F | caatgtatccttactggcttctc | The promoter of acnA: 231 bp |
| acnA-R | tgccgtctaatctcgtcaaa |  |
| ompF(FAM)-F | acaacggttctacaagccaata | The promoter of ompF: 221 bp |
| ompF-R | gtgtctgacaggcatctttcca |  |
| PlaczompF-F | gaagatcttgaaatagctcttctgctgc | The promoter of *ompF* with *Bgl* Ⅱ and *Nco* Ⅰ sites: 344 bp |
| PlaczompF-R | catgccatggtatttattaccctcattggtt |  |
| PlaczacnA-F | gaagatctattaaacacagttggtgtcgc | The promoter of *acnA* with *Bgl* Ⅱ and *Nco* Ⅰ sites: 288 bp |
| PlaczacnA-R | catgccatggagctcctccttaaatgacag |  |
| PlaczphoP-F | gaagatctaactgaaagagttgacccg | The promoter of *acnA* with *Bgl* Ⅱ and *Nco* Ⅰ sites: 284 bp |
| PlaczphoP-R | catgccatggctcttctcccttgtgtta |  |
| ompF-F | tctgacttctttggtctggtgg | The fragment of *ompF*：166 bp |
| ompF-R | gcttgctgttgctgtacgctg |  |
| acnA-F | gcattcagccgtttcagcg | The fragment of *acnA*：114 bp |
| acnA-R | aagcaatccactccccgtc |  |

**Reference**

1. Zhang Z, Chang J, Xu X, Zhou M, Shi C, Liu Y, Shi X. 2021. Dissemination of IncFII plasmids carrying fosA3 and blaCTX-M-55 in clinical isolates of *Salmonella Enteritidis*. Zoonoses Public Health 68:760-768.
2. Hu M, Huang X, Xu X, Zhang Z, He S, Zhu J, Liu H, Shi X. 2022. Characterization of the role of two-component systems in antibiotic resistance formation in Salmonella enterica serovar Enteritidis. mSphere ;e0038322.

3. Bi D, Jiang X, Sheng ZK, Ngmenterebo D, Tai C, Wang M, Deng Z, Rajakumar K, Ou HY. 2015. Mapping the resistance-associated mobilome of a carbapenem-resistant *Klebsiella pneumoniae* strain reveals insights into factors shaping these regions and facilitates generation of a ‘resistance-disarmed’ model organism. J Antimicrob Chemother 70:2770-4.

4. Qian H, Yu H, Li P, Zhu E, Yao Q, Tai C, Deng Z, Gerdes K, He X, Gan J, Ou HY. Toxin-antitoxin operon kacAT of Klebsiella pneumoniae is regulated by conditional cooperativity via a W-shaped KacA-KacT complex. Nucleic Acids Res. 2019 Aug 22;47(14):7690-7702.
