# Supplementary material for: PhoPQ Regulates Quinolone and Cephalosporin Resistance Formation in Salmonella Enteritidis at the Transcriptional Level
Source: mBio. 2023 May 15;14(3):e03395-22. doi: 10.1128/mbio.03395-22 (PMC10294627; doi:10.1128/mbio.03395-22)
Supplement: TABLE S2 [file mbio.03395-22-s0010.docx]

| Gene | E | R^2^ | Gene | E | R^2^ |
| --- | --- | --- | --- | --- | --- |
| *phoQ* | 1.05 | 0.997 | *csrA* | 1.04 | 0.997 |
| *gyrB* | 1.04 | 0.997 | *sirC* | 1.01 | 0.990 |
| *gcvT* | 0.99 | 0.996 | *osmY* | 1.05 | 0.995 |
| *plsC* | 0.93 | 0.993 | *ompF* | 1.01 | 0.990 |
| *ycgK* | 1.04 | 0.986 | *traE* | 1.03 | 0.985 |
| *rbsK* | 1.05 | 0.992 | *acnA* | 1.05 | 0.998 |
| *nadB* | 0.99 | 0.992 | *vanX* | 1.05 | 0.988 |
| *exuT* | 1.10 | 0.986 | *nadE* | 1.05 | 0.988 |
| *DXN21-RS14530* | 0.95 | 0.983 | *cfa* | 1.05 | 0.997 |
| *kduI* | 0.98 | 0.985 | *frmA* | 1.01 | 0.973 |
| *DXN21-RS19045* | 0.97 | 0.992 | *DXN21-RS18010* | 1.05 | 0.997 |
| *flhD* | 1.07 | 0.998 | *DXN21-RS20320* | 1.02 | 0.998 |
| *dsmA* | 1.05 | 0.982 | *tyrR* | 1.03 | 0.997 |
| *spvA* | 1.04 | 0.998 | *RS4885* | 1.02 | 0.985 |
| *slyB* | 1.05 | 0.997 | *mgtA* | 1.02 | 0.998 |
| *mucR* | 1.05 | 0.989 | *DXN21-RS12245* | 0.95 | 0.984 |
| *DXN21-RS22435* | 0.99 | 0.998 | *DXN21-RS05075* | 1.00 | 0.999 |
| *malK* | 0.98 | 0.997 | *yrbN* | 1.02 | 0.995 |
| *ftsL* | 0.99 | 0.998 | *pgtE* | 1.03 | 0.998 |
| *ramA* | 0.96 | 0.999 | *lexA* | 1.00 | 0.999 |
| *marR* | 0.99 | 0.998 | *ssaG* | 0.99 | 0.995 |
| *DXN21-RS04885* | 1.02 | 0.996 | *eamB* | 1.05 | 0.999 |
| *sscA* | 0.99 | 0.998 | *invF* | 1.00 | 0.995 |
| *sseE* | 0.99 | 0.977 | *pagP* | 1.02 | 0.997 |
| *yrbL* | 0.99 | 0.998 | *sseA* | 0.99 | 0.986 |
