# Supplementary material for: PhoPQ Regulates Quinolone and Cephalosporin Resistance Formation in Salmonella Enteritidis at the Transcriptional Level
Source: mBio. 2023 May 15;14(3):e03395-22. doi: 10.1128/mbio.03395-22 (PMC10294627; doi:10.1128/mbio.03395-22)
Supplement: TEXT S1 [file mbio.03395-22-s0001.doc]

1. **Scanning electron microscope (SEM) observation**

Cell morphology of *S.* Enteritidis and Δ*phoP* strains without or with antibiotics treatment were observed by SEM as previously described (1).

1. **Membrane fatty acid analysis**

Extraction of membrane fatty acid methyl esters was carried out according to the method described by Sasser *et al*. and Qin *et al*.(1,2), with a slight modification. The lipids were enriched with 1 mL saponification reagent from 40 mg of each pelleted cells, and then lipids were methylated in an acid medium using methanol and 6N hydrochloric acid; afterwards, lipids were extracted using [hexane](javascript:;) and methyl tert-butyl ether (1:1 v/v) solution and rotated for 10 min to obtain the organic phase; in the last step, 3 mL alkaline lye and 500 μL of saturated NaCl solution were added into each tube and tumbled for 3 min. Subsequently, two-thirds of the organic phase was pipetted into a GC vial and capped.

The fatty acid methyl esters were analyzed using a 7890A Gas-Chromatography instrument and identified by comparing retention times with those of standards, MIDI calibration mix (MIDI). Calibration curves (R^2^ > 0.99) of the peak area against the fatty acid methyl ester concentration were constructed to determine the concentration of the fatty acid methyl esters. Three independent experiments were completed. The results were expressed as the relative percentage of individual fatty acid, which was calculated as the molar ratio of the fatty acid to all fatty acids.

1. **Membrane perturbation assay**

The 1-N-phenylnaphthylamine (NPN) uptake assay was performed to determine the outer membrane permeability (3). NPN was added to the culture containing 2 × 10^6^ CFU/mL *S*. Enteritidis wild-type and Δ*phoP* cells (final NPN concentration of 12.5 μM) and incubated for 15 min with varying concentrations of antibiotics. The fluorescence emission intensity was recorded (λ_exc_ = 340 nm, λ_em_ = 420 nm, gap width 1 mm) using an Infinite 200 PRO [microplate](javascript:;) [reader](javascript:;) (Tecan, Switzerland).

1. **Lipopolysaccharide extraction**

The lipopolysaccharide (LPS) Extraction kit (iNtRON Biotechnology, Korea) was used following the manufacturer’s instruction. The extrated LPS samples were separated on 15% SDS-PAGE (Bio-Rad Laboratories, USA), and fixed 1 h in buffer with 0.1% formaldehyde, 12% acetic acid and 50% ethanol. The gels were stained in 0.5% AgNO_3_ and 0.075% formaldehyde solution, and colored in buffer with 6% Na_2_CO_3_, 0.004% sodium thiosulfate and 0.05% formaldehyde. All the solutions were prepared fresh before use.

1. **Reactive oxygen species measurement**

The intracellular ROS were measured using an ROS Assay Kit (Beyotime, Shanghai, China). The exponentially growing bacterial samples were collected by centrifugation and were treated with 2′,7′-dichlorofluorescein diacetate for 40 min in 37 °C at the dark. The [fluorescence intensity](https://www.sciencedirect.com/topics/chemistry/fluorescence-intensity) values (λ_exc_ = 488 nm, λ_em_ =525 nm) were recorded at 0 h, 2 h and 8 h after antibiotics incubation with *S.* Enteritidis and Δ*phoP* at 37℃. A similar procedure was also used for the measurement of ROS in a cell-free system as quality control.

1. **Intracellular NAD^+^/NADH quantification**

Intracellular concentrations of NAD^+^ and NADH in WT and Δ*phoP* were measured using NAD+/NADH Assay Kit with WST-8 (Beyotime, Shanghai, China). Briefly, cells untreated and treated with antibiotics for 8 h were harvested by centrifugation (12,000 rpm, 3 min, 4℃) and resuspended in 400 μL extracting buffer to obtain the total of NAD^+^ and NADH. A half portion of the extract was heated at 60℃ for 30 min to degrade NAD^+^ until only NADH remained. Then alcohol dehydrogenase was added to the pure NADH extracts and the rest extracts, all extract samples were placed at 37℃ for 10 min to convert NAD^+^ into NADH. Finally, the color reagent was used to quantify the NADH concentrations at the wavelength of 450 nm. According to a range of NADH standards (0–200pM), NADH concentrations in the cell extracts were determined, while the NAD^+^ concentrations were equal to the total of NAD^+^ and NADH minus pure NADH.

1. **Enzyme activity assay**

The enzyme activity of catalase (CAT) and superoxide dismutase (SOD) were measured using Catalase Assay kit and Superoxide Dismutase Assay Kit with WST-8 ((Beyotime, Shanghai, China) respectively. Briefly, 1mL cells of WT and Δ*phoP* untreated and treated with antibiotics for 8 h were harvested and washed with PBS. The suspended cells were disrupted by sonification on ice at 100 W for 12 times at 5-sec intervals, and then cell debris were removed. For CAT activity measurement, the cell extracts were mixed with work solution, followed by adding H_2_O_2_. After incubation for 2 min, stop solution and peroxidase reagents were added in turn to decompose the remained H_2_O_2_ along with the production of a red substance, N-(4-antipyryl)-3-chloro-5-sulfonate-pbenzoquinonemonoimine, which could be determined at the wavelength of 520 nm. For the activity of SOD determined, WST-8 reacts with superoxide radical anion (O^2−^ ) to produce the water-soluble formazan dye. This reaction can be suppressed by SOD catalysis that converts O^2−^ to H_2_O_2_ and O_2_. Thus, the production of formazan dye measured at the wavelength of 450 nm is negatively correlated with SOD activity. To normalize the enzyme activity assay, the concentration of total protein in crude cell extract was determined using BCA Protein Quantification Kit (Yeasen, Shanghai, China).

1. **Bacterial genetic manipulations**
2. Gene deletion and complementation

The Δ*phoQ* was generated by a flippase recognition target site-flanked *hph* cassette via homologous recombinase as described previously (4). The PhoQ (GenBank: TBS05337.1) gene was amplified from the whole genome cDNA of *S.* Enteritidis with C-terminally 6×His and two restriction sites *EcoR* I and *Xba* I; then, the fragment was introduced into the low-copy vector pBAD33-Apra plasmid (pBAD33-*hisphoQ*) with the arabinose inducible promoter, and further transferred into Δ*phoQ* to obtain the complementary strain Δ*phoQ*-*hisphoQ*. The strains, vectors and primers used in this study were listed in Table S1 and Table S2.

1. Recombinant expression of PhoP and truncated PhoQ

The open reading frame of *phoP* and *phoQ* sensor domain (PhoQ^SD^) sequences were amplified and then cloned into pET28a with *Nde* I-*Xho* I or *Nco* I-*Xho* I pair sites to generate the expression plasmid pET28a-*hisphoP* (theoretical molecular weight 27.8 kDa) and pET28a-*hisphoQ*^SD^ (theoretical molecular weight 18.55 kDa). The final plasmid was confirmed by sequencing. The N-terminus His-tagged PhoP protein and C-terminus His-tagged PhoQ^SD^ protein were purified from the cell-lysis supernatant of *E. coli* BL21(DE3) via His-Select nickel affinity gel (Ni-NTA beads 6FF, Smart-Lifesciences), respectively. The recombinant proteins were determined by Western-Blot using anti-His antibody. All proteins were desalted and concentrated using a 10-kDa Millipore ultracentrifugally filter at 4°C. The concentration of protein was determined using BCA kit and stored at –80°C before use.

1. Site-directed mutagenesis

Point mutations were made by QuickMutation™ Site-Directed Mutagenesis Kit (Beyotime, Shanghai, China). In briefly, the specific site-directed mutagenesis primers contained the mutation sites of D45A/K46Q, T48A/R50L and K186Q/R187L/S188A were designed (Table S2) and used to amplify the plasmid of pBAD33-*hisphoQ* via BeyoFusion™ DNA Polymerase, whereas the unamplified plasmids would digest by *Dpn* I. The PCR products were purified using the universal DNA purification kit and connected with pBAD33 via restriction sites *EcoR* I and *Xba* I. The obtained plasmid pBAD33-*hisphoQ*^mutant^ was transferred to *S.* Enteritidis Δ*phoQ* strain to create variants of PhoQ (named Δ*phoQ*-*hisphoQ*^mutant^) (Table S1).

Above mentioned mutants, variants, plasmids and PCR products were confirmed by sequencing (Sangon, Shanghai, China).

1. **Electrophoretic mobility shift assay (EMSA)**

Purified His-tagged PhoP proteins were phosphorylated using acetyl phosphate lithium potassium salt similar as previously described (5-7). Briefly, PhoP was phosphorylation by 50 mM acetyl phosphate lithium potassium salt in a reaction buffer containing 10 mM Tris-HCl (pH 8.0), 2 mM MgCl_2_, 125 mM KCl, 1 mM EDTA, and 5% glycerin at 30°C for 2 h. A FAM-labeled *acnA* promoter probe (P*_acnA_*-FAM, 231bp) and *ompF* promoter probe (P*_ompF_*-FAM, 211bp), or unlabeled gene promoter probe (P*_acnA_* and P*_ompF_*) were generated by PCR amplification, and then the probe was purified using a AxyPrep DNA Gel extraction kit. The primer sequences were listed in Table S2.

The binding reactions were performed in a 20-μL system containing 10 mM Tris (pH 8.0), 125 mM KCl, 5% glycerin, 2 mM MgCl_2_, 0.01 mg/mL bovine serum albumin, 1 mM dithiothreitol, 1~8 μg phosphorylated PhoP (PhoP-P), 25 nM FAM-labeled probes with or without 1,250 nM unlabeled competitor probe, and 0.5 μg of poly(dI-dC). The reaction systems were incubated at 30°C for 30 min. Native polyacrylamide gels (5%) were pre-electrophoresed for 1 h, then the samples were loaded and separated by electrophoresis on the 4℃. The fluorescent migration stripes of nucleic acid were scanned using a ChemiDoc touch imaging system (Bio-Rad, Hercules, CA).

1. **Analysis of *ompF* and *acnA* promoters in different species**

The promoter region of *ompF* and *acnA* (*DXN21_RS15940* and *DXN21_RS05400*) in SJTUF 12367 and its homologous genes from different species, including STM0999 and STM1712 from *Salmonella enterica* serovar Typhimurium strain LT2 (GenBank accession number AE006468.2), AW48_15445 and AW48_11290 from *Salmonella enterica* subsp. enterica serovar Newport strain CDC 2012K-0938 (GenBank accession number CP025246.1), DZA54_15925 and DZA54_13155 from *Salmonella enterica* subsp. enterica serovar Dublin strain USMARC-69807 (GenBank accession number CP032379.1), HEC76_14590 and HEC76_11115 from *Salmonella enterica* subsp. enterica serovar Indiana strain SI67 (GenBank accession number CP050783.1), GJE06_14280 and GJE06_10660 from *Salmonella enterica* serovar Birkenhead strain AUSMDU00010532 (GenBank accession number CP045958.1), DOE61_15145 and DOE61_12645 from *Salmonella enterica* serovar Berta strain SA20141895 (GenBank accession number CP030005.1), D8B36_14430 and D8B36_12585 from *Escherichia coli* strain K-12 substrain MG1655 (GenBank accession number CP032667.1), SBG_0853 and SBG_1575 from *Salmonella* *bongori* NCTC 12419 (GenBank accession number CP053416.1), SF0926 and SF1280 from *Shigella flexneri* 2a strain 301 (GenBank accession number AE005674.2), CKO_02137 and CKO_01358 from *Citrobacter koseri* strain ATCC BAA-895 (GenBank accession number CP000822.1), were obtained from the National Center for Biotechnology Information and aligned using the ClustalW (http://www.ebi.ac.uk/Tools/msa/clustalw2), and the region with the identified PhoP binding site was illustrated.

1. **Promoter analysis by *lacZ* fusions**

To generate the *lacZ* reporter plasmid, the 284-bp *phoP* promoter, 344-bp *ompF* promoter, and 288-bp *acnA* promoter DNA were inserted upstream of the *lacZ* gene of promoterless plasmid pLACZ using *Bgl* Ⅱ and *Nco* Ⅰ restriction enzyme sites, resulting in the fusion plasmid pLACZ-P*_phoP_*, pLACZ-P*_ompF_* and pLACZ-P*_acnA_*, respectively. In addition, the full-length *phoP* gene was PCR-amplified from SJTUF12367 genomic DNA and cloned into an arabinose-inducible vector pBAD33 to construct the PhoP constitutive expression plasmid pBAD33-*phoP*. These fused plasmids and pBAD33-*phoP* were transferred into *E. coli* DH5α strain. The positive clones were grown in LB supplemented with 0.2% arabinose for 5 h to induce PhoP. β-Galactosidase activity was then determined according to the standard Miller method, using SDS and chloroform to permeabilize the cells (8).

1. **Phosphorylation Assay**

Protein phosphorylation was performed using a Phos-tag (9). For *in vivo* phosphorylation, Δ*phoQ*-*hisphoQ* and Δ*phoQ*-*hisphoQ*^mutant^ were grown in LB with or without nalidixic acid (32 μg/mL) and ceftazidime (2 μg/mL) pressure. Following 3 h of treatment, whole-cell lysate was obtaining by sonication and resolved in 8% SDS gels (29:1) containing 50 μM Phos-tag acrylamide (Wako) and 100 μM MnCl_2_. Proteins in the Phos-tag gels were transferred onto a nitrocellulose membrane and determined by western-blot using anti-His antibody. For *in vitro* phosphorylation, *E.coli* strain harboring pBAD33-*hisphoQ* were grown in LB at 37°C, and the expression of PhoQ was induced by 0.2% arabinose for 4 h with shaking. Following centrifugation (5,000×g) for 5 min, and the Bacterial Membrane Protein Extraction Kit (Bestbio, China) was used to obtain the crude membrane extracts contained His-tagged PhoQ protein. The extracted protein was treated with different antibiotics of nalidixic acid, [ciprofloxacin](javascript:;), [ofloxacin](javascript:;), ceftazidime, [ceftriaxone](javascript:;), [cefepime](javascript:;), colistin, and sulfisoxazole. Subsequently, the samples were resolved in Phos-tag gels, and protein mobility shift was further determined.

1. **Differential scanning calorimetry (DSC) analysis**

The 800 μg/mL PhoQ^SD^ protein (~41.3 μM) was incubated with nalidixic acid/ceftazidime (100 μM) antibiotics in PBS pH 7.4 or pH 5.5 for 1 h. Then the pure PhoQ^SD^ protein and incubated samples were degassed and loaded in NanoDSC-6300 DSC (TA Instruments, America). Set the running parameters from 5°C to 95°C with a heating/cooling rate of 1°C/min on 0.9 mL sample. DSC curve analysis was worked out using Launch NanoAnalyze software.

1. **Microscale thermophoresis (MST) analysis**

The binding affinity of the PhoQ^SD^ with antibiotics was measured by Monolith NT.115 (Nanotemper Technologies) (10). The proteins used were desalted to MST buffer (PBS-0.05% tween, pH 7.4) and labeled with a fluorescent dye RED-tris-NTA 2nd Generation (Nanotemper Technologies) reactive on [histidine](javascript:;). For each assay, an equal volume of the serial dilution antibiotics (nalidixic acid, ceftazidime, [azithromycin](javascript:;), colistin, sulfamethoxazole and ofloxacin) and of the constant labeled proteins (100 nM) were mixed and incubated for 30 min at dark. The samples were then loaded in glass capillaries (NanoTemper Technologies) and measured at 25 °C by using 80% LED power and medium MST power. The dose response curve was plot based on three biological replicates and analyzed using MO.Affinity Analysis v.2.3 software, which set *Kd* fit model for calculate *Kd* value.

1. **Homology modeling and molecular docking**

In view of the unavailable for complete structure of PhoQ protein and the HK usually sensor the environment signal in the cell periplasmic with dimer status, the crystal structure of *E.coli* and *S.* Typhimurium PhoQ sensor domain (PDB ID:3BQ8 and 1YAX) were selected for PhoQ or PhoQ^mutant^ homologous modeling through SWISS-MODEL (<http://swissmodel.expasy.org/>), with a sequence identity exceed 81.08%.

For docking analysis, the three-dimensional structure of ligands (nalidixic acid and ceftazidime) was downloaded from the PubChem database in SDF format, whose energy was minimized using ChemDraw. The file formats of the ligands were converted to the PDBQT format via AutoDock 4.2. The hydrogens must be added to the ligands and the protein, whereas non-polar hydrogens were removed. The docking on the dimer model of PhoQ^SD^ with ligands were performed using AutoDock 4.2, which is based on Lamarckian Genetic Algorithm, a hybrid genetic algorithm with local optimization that uses a parameterized free energy scoring to estimate the binding energy. The three-dimensional structures of the complexes were generated in the PDB format according to the lowest binding energy and was presented using Discovery Studio 4.0 client software (11).

**References**

1. Xiaojie Qin, Rui Dong, Shoukui He, Xiujuan Zhou, Zengfeng Zhang, Yan Cui, Chunlei Shi, Yanhong Liu, Xianming Shi. 2020. Characterization of the role of ybgC in lysozyme resistance of Salmonella Enteritidis. Food Control 109:106732.
2. Sasser, M. 2001. MIDI technical note #101: Bacterial identification by gas chromatographic analysis of fatty acids methyl esters (GC-FAME). Netwark, Delaware: MIDI, Inc.
3. Elliott AG, Huang JX, Neve S, Zuegg J, Edwards IA, Cain AK, Boinett CJ, Barquist L, Lundberg CV, Steen J, Butler MS, Mobli M, Porter KM, Blaskovich MAT, Lociuro S, Strandh M, Cooper MA. 2020. An amphipathic peptide with antibiotic activity against multidrug-resistant Gram-negative bacteria. Nat Commun 23;11(1):3184.
4. Bi D, Jiang X, Sheng ZK, Ngmenterebo D, Tai C, Wang M, Deng Z, Rajakumar K, Ou HY. 2015. Mapping the resistance-associated mobilome of a carbapenem-resistant *Klebsiella pneumoniae* strain reveals insights into factors shaping these regions and facilitates generation of a ‘resistance-disarmed’ model organism. J Antimicrob Chemother 70:2770-2774.
5. Hellman LM, Fried MG. 2007. Electrophoretic mobility shift assay (EMSA) for detecting protein-nucleic acid interactions. Nat Protoc 2(8):1849-61.
6. Huang X, Hu M, Zhou X, Liu Y, Shi C, Shi X. 2020. Role of *yoaE* Gene Regulated by CpxR in the Survival of Salmonella enterica Serovar Enteritidis in Antibacterial Egg White. mSphere 5(1):e00638-19.
7. Arnett KL, Blacklow SC. 2014. Analyzing the nuclear complexes of Notch signaling by electrophoretic mobility shift assay. Methods Mol Biol 1187:231-45.
8. Griffith KL, Wolf RE. 2002. Measuring β-Galactosidase activity in Bacteria: Cell growth, permeabilization, and enzyme assays in 96-Well arrays. Biochem. Biophys. Res. Commun., 290, 397-402.
9. Li L, Wang Q, Zhang H, Yang M, Khan MI, Zhou X. 2016. Sensor histidine kinase is a β-lactam receptor and induces resistance to β-lactam antibiotics. Proc Natl Acad Sci U S A 9;113(6):1648-53.
10. Zhang N, Liu Y, Shi X, Zhang Y, Li W, Yang Y, Chen L, Yin Y, Tong L, Yang J, Luo J. 2022. Microscale thermophoresis and fluorescence polarization assays of calcineurin-peptide interactions. Anal Biochem 646:114626.
11. Saral Sariyer A. 2022. Three new inhibitors of class A β-lactamases evaluated by molecular docking and dynamics simulations methods: relebactam, enmetazobactam, and QPX7728. J Mol Model 28(4):76.
